# Microbial competition for iron determines its availability to the ferrous wheel

**DOI:** 10.1093/ismejo/wraf015

**Published:** 2025-01-27

**Authors:** Robert F Strzepek, Pauline Latour, Michael J Ellwood, Yeala Shaked, Philip W Boyd

**Affiliations:** Australian Antarctic Program Partnership, Institute for Marine and Antarctic Studies, University of Tasmania, 20 Castray Esplanade, Hobart, TAS 7004, Australia; ARC Australian Centre for Excellence in Antarctic Sciences, University of Tasmania, Hobart, TAS 7004, Australia; Institute for Marine and Antarctic Studies, University of Tasmania, Hobart, TAS 7004, Australia; Research School of Earth Sciences, Australian National University, Canberra, ACT 0200, Australia; The Fredy and Nadine Herrmann Institute of Earth Sciences, Edmond J. Safra Campus, Givat Ram, Hebrew University of Jerusalem, Jerusalem 9190401, Israel; The Interuniversity Institute for Marine Sciences in Eilat, Eilat 8810302, Israel; Australian Antarctic Program Partnership, Institute for Marine and Antarctic Studies, University of Tasmania, 20 Castray Esplanade, Hobart, TAS 7004, Australia; Institute for Marine and Antarctic Studies, University of Tasmania, Hobart, TAS 7004, Australia

**Keywords:** iron, carbon, Southern Ocean, microbes, heterotrophic bacteria, picoeukaryote, phytoplankton, competition, light, ferrous wheel

## Abstract

Iron plays a pivotal role in regulating ocean primary productivity. Iron is supplied from diverse sources such as the atmosphere and the geosphere, and hence iron biogeochemical research has focused on identifying and quantifying such sources of “new” iron. However, the recycling of this new iron fuels up to 90% of the productivity in vast oceanic regions. Evidence points to the key role of microbes in mediating this recycling, referred to as the “ferrous wheel”, that remobilises iron initially supplied to ocean biota. In the iron-limited subantarctic waters of the Southern Ocean, iron uptake is dominated by microbes smaller than 2 μm and exhibits seasonal and depth-related variations. The microbial community within the <2 μm size fraction comprises heterotrophic bacteria and picophytoplankton, both competing for iron. Here, we dissect the demand component of the ferrous wheel by separately assessing iron uptake by heterotrophic bacteria and photoautotrophic picophytoplankton. To explore the seasonal and depth-related variability in iron uptake, the influence of light on iron uptake in both bacterial and phytoplankton communities was examined. We observed that picoeukaryote phytoplankton demonstrated iron uptake rates 10 times greater than those observed in bacteria when normalized to biomass. Light was shown to stimulate iron uptake by 8- to 16-fold in phytoplankton and by 4- to 8-fold in heterotrophic bacteria. These results highlight the unexpectedly significant role of picoeukaryotic phytoplankton in driving the speed of the ferrous wheel, with implications for iron recycling across diurnal cycles, different oceanic depths, and seasonally.

## Introduction

The trace element iron (Fe) plays a central role in the biogeochemical cycles of key elements such as carbon, oxygen, nitrogen, sulfur, and phosphorus through its regulation of key microbial physiological rates including photosynthesis and respiration [1]. Despite being one of the most abundant elements in the Earth’s crust, Fe is present at vanishingly low concentrations over much of the global ocean due to both its limited solubility and from being extremely particle reactive [2]. Such reactivity means that Fe is readily removed from the water column in nearshore waters [3]. Despite dissolved Fe being present at picomolar concentrations in much of the surface ocean, these concentrations are typically higher than those set by the solubility limit of inorganic Fe in seawater due to organic complexing agents, called ligands, which are often produced by microbes and enhance Fe solubility [2]. Ligands bind over 99% of dissolved Fe in seawater and largely control the bioavailability of Fe to microorganisms. The bioavailability of Fe is further affected by photochemical reactions that make Fe complexed to ligands more available for uptake [2].

The pivotal role of Fe in cellular physiology along with the low availability of dissolved Fe means that it is an element that is “heavily trafficked” by ocean biota [2]. Fe is supplied by wide-ranging mechanisms such as aerosol dust, ocean upwelling and melting icebergs [4]. Most research on the marine biogeochemistry of Fe since the 1990s has focused on quantifying these “new” sources of Fe for example hydrothermal inputs [5]. In contrast, the role of recycled Fe is understudied. Iron recycling is known to play a central role in the terrestrial biosphere where it is termed the “ferrous wheel” [6]. In the ocean, the ferrous wheel is comprised of the key microbes—bacteria, phytoplankton, viruses, and grazers—contributing to Fe demand and supply [4]. Only a few studies have quantified the relative contribution of new versus recycled Fe. The major contribution of recycled Fe results in a very low Fe-ratio (i.e. new Fe uptake/uptake of new and recycled Fe) [7, 8]. Process studies show seasonality in the Fe-ratio: ~0.1 (i.e. 90% of Fe for biota comes from recycling) during summer in low Fe High Nitrate Low Chlorophyll (HNLC) subantarctic waters [7]; ~0.6 to 0.1 during a subtropical springtime bloom [9, 10]. The patterns observed in these two short-term process studies were extended to estimate the relative contributions of new and recycled Fe to the Southern Ocean over the seasonal cycle [5]. Subsequent modelling has suggested that Fe recycling is central to driving annual primary productivity but there are currently few data available to improve model parameterizations [11, 12].

Previous results from the subantarctic Southern Ocean Time Series (SOTS) site in the fall showed that cells <2.0 μm in diameter take up most of the Fe [13]. This size class comprises heterotrophs (bacteria) and photoautotrophs (phytoplankton) that compete for this limiting resource [14, 15]. Therefore, we sought to answer the following question: how much Fe do heterotrophic bacteria take up compared to phytoplankton? Answering this question is important because the fate of Fe (recycled versus exported) and its efficiency in fuelling primary production depend on whether bacteria or phytoplankton take it up [16]. However, competition is just one aspect of this narrative, as bacteria also rely on labile dissolved organic carbon (DOC) released by phytoplankton as a carbon (C) source and microbes release Fe-binding ligands that can facilitate Fe uptake by both bacteria and phytoplankton [17–21].

**Figure 1:**
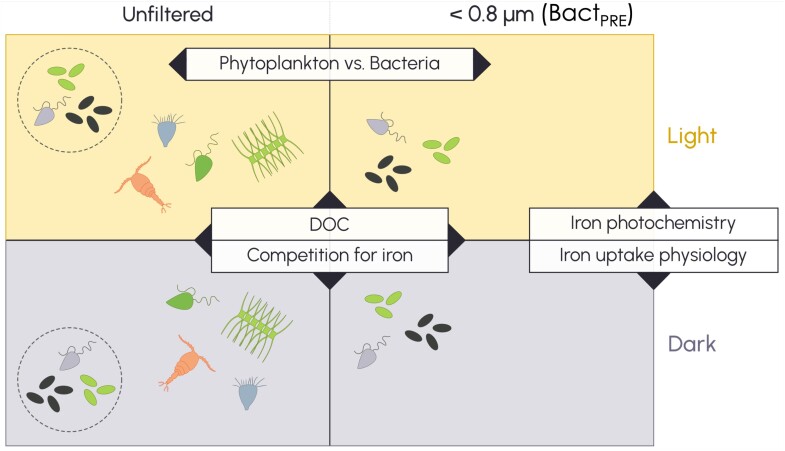
Conceptual illustration of the ferrous wheel size-fractionation experimental design. Treatments were unfiltered and pre-incubation <0.8 μm filtrations (Bact_PRE_) incubated in the light and in the dark. Pre-incubation size fractionation (Bact_PRE_) isolated heterotrophic bacteria from the ferrous wheel. Comparison with post-incubation size fractionation allowed us to compare the effects of isolating heterotrophic bacteria from the ferrous wheel and to compare the effects of light and dark treatments on Fe uptake by both phytoplankton and heterotrophic bacteria. Dashed circles represent the <0.8 μm (0.2–0.8 μm) fraction isolated from the unfiltered treatments during post-incubation size fractionation. Labelled boxes denote processes or groups affected by the treatments.

The biogeochemical cycles of Fe and C are linked by the Fe:C ratio—as an elemental or, in our study, uptake ratio—in phytoplankton and heterotrophic bacteria. We have observed that Fe uptake is less sensitive to changes in underwater irradiance than inorganic C uptake is (i.e. net primary productivity, NPP) [13, 22]. This suggests that either (i) phytoplankton Fe uptake is less sensitive to changes in irradiance than is NPP; or (ii) a process less sensitive to light, such as heterotrophic bacterial assimilation, is responsible for most of the Fe uptake. We thus asked how light affects Fe uptake in bacteria and phytoplankton. Possible light-mediated influences include photochemical reactions that make Fe complexed to ligands more available for uptake in the light, and/or the regulation of physiological processes that influence Fe uptake. In the latter case, light might affect Fe uptake directly by supplying reductant and/or energy produced photosynthetically for extracellular Fe reduction and uptake, or indirectly, such as through altering the supply of DOC to fuel the growth of heterotrophic bacteria (Fig. 1).

Here, we dissect the spokes of the ferrous wheel associated with Fe demand by quantifying the uptake rates of heterotrophic bacteria and phytoplankton in the subantarctic Southern Ocean during summer and situate these findings within a seasonal context. To do so, we conducted bioassays in which the effects of light on Fe photochemistry and uptake physiology were studied by comparing light and dark incubations, and the effects of DOC supply and competition between phytoplankton and heterotrophic bacteria were examined by isolating bacteria from the larger members of the ferrous wheel by pre-incubation size fractionation (Fig. 1).

## Materials and methods

Voyage information. Experiments were performed onboard the RV *Investigator* during three voyages, IN2018_V04 in September 2018 (austral spring), IN2018_V02 in March 2018 (austral fall) and IN2020_V08 in December 2020 (austral summer) at the SOTS site (46.80°S, 141.884°E). This site is within the subantarctic zone to the southwest of Tasmania, Australia and has been the focus of prior Fe biogeochemical studies [13, 23].

CTD Sampling. Prior to starting the experiments, initial oceanographic settings were studied at the SOTS site through the deployment of a 36-bottle conductivity-temperature-depth (CTD) rosette. Profile data were collected with a winch-lowered package consisting of an SBE 911plus CTD equipped with an array of sensors to measure oxygen, fluorescence, and photosynthetically active radiation (PAR; Sea-Bird Electronics sensors: SBE4C, SBE3 T, SBE9plus, SBE43, FLBBNTU, QCP–2300 HP; Bellevue, WA, USA).

Trace metal-clean water sampling. Seawater samples for trace metal-clean incubations were collected using Teflon-coated 12-L Niskin bottles attached to an autonomous rosette equipped with a CTD unit (SeaBird 911 plus). Upon retrieval, the Niskin bottles were transferred into an ISO Class 5 containerized clean room. Seawater samples for dissolved trace metal analysis were filtered through acid-cleaned 0.2-μm capsule filters (Supor AcroPak 200, Pall, Port Washington, NY, USA) and acidified with distilled nitric acid to a final pH ≤ 1.8. The sampling protocols followed recommendations in the GEOTRACES Cookbook (https://www.geotraces.org/methods-cookbook/). Samples for flow cytometry and fast repetition rate fluorometry (FRRF) were collected from the trace metal rosette to characterise the initial plankton communities.

Iron uptake and NPP simulated *in situ* depth profiles. Net primary productivity (i.e. inorganic C uptake) and Fe uptake rates were determined for water column samples collected at four to six depths between 0 and 70 m with the trace metal rosette. Water samples were collected pre-dawn at sampling depths determined from *in situ* PAR depth profiles obtained during midday CTD casts the day prior to collection. Samples were dispensed into 300 mL acid-washed polycarbonate bottles (ThermoScientific Nalgene, Waltham, MA, USA) and spiked with 16–20 μCi of sodium ^14^C-bicarbonate (NaH^14^CO3; specific activity 1.85 GBq mmol^−1^; PerkinElmer, Waltham, MA, USA) and 0.2 nmol L^−1^ of an acidified ^55^Fe solution (^55^FeCl_3_ in 0.1 M Ultrapure HCl; specific activity 30 MBq mmol^−1^; PerkinElmer). Six samples (five light and one “dark correction” bottle per irradiance) were incubated for 24 hours in a temperature-controlled deckboard incubator under natural sunlight at four to six light intensities (ranging from 67 to 1.0% of incident PAR). Light attenuation was adjusted by varying the layers of neutral density mesh and measured with a Biospherical Instruments QSL2101 Quantum Scalar PAR Sensor (San Diego, CA, USA). The incubation temperatures were 9, 10 and 12 ± 0.2°C for the spring, summer and fall experiments, respectively.

After 24 hours of incubation, four replicate samples were serially vacuum-filtered (<10 mm Hg) through 20, 2.0, and 0.2 μm porosity polycarbonate filters (47 mm diameter; Poretics, Waltham, MA, USA) separated by 200 μm nylon mesh spacers to determine size-fractionated Fe uptake rates and NPP. Two size-fractionated samples were washed with Titanium (III) EDTA—citrate reagent for 5 min to remove extracellular Fe [24] and rinsed three times with 15 mL of 0.2 μm-filtered seawater, and the other two size-fractionated samples were rinsed only with 0.2 μm-filtered seawater. In addition, two samples were filtered through 0.2 μm filters: an unfractionated light measurement and a dark correction bottle. The dark correction was 1.6 ± 1.4% of the C uptake rates measured in the light over multiple voyages, depths, and years (*N* = 11). Data for the dark-corrected, size-fractionated samples rinsed with the Ti(III) EDTA— citrate reagent are reported here (i.e. intracellular Fe and C uptake) (*N* = 2). The one exception to this protocol was for the size-fractionated depth profile samples collected from 19 m in summer 2020 that were performed in triplicate and incubated in parallel with the ferrous wheel experiment (*N* = 3). Samples were counted on a liquid scintillation counter (PerkinElmer Tri-Carb 2910 TR) as described previously [13]. Dissolved Fe concentrations were measured through high-resolution inductively coupled plasma mass spectrometry (HR-ICP-MS; Element XR; ThermoScientific) after pre-concentration at the Australian National University (Canberra, Australia). The detection limit was 0.06 nmol L^−1^ for dissolved Fe (*N* = 3) [25].

Ferrous wheel experiment. The ferrous wheel experiment was conducted in summer 2020 (IN2020_V08) using seawater from the same sampling bottle of the trace metal rosette as the 19 m treatment of the 24 hours simulated *in situ* depth profile incubation (Fig. S1). We exploited the difference in size between heterotrophic bacteria and photoautotrophic phytoplankton to separate these groups using acid-washed polycarbonate membrane filters of appropriate porosity (0.8 μm). We performed a pre-incubation filtration to isolate the heterotrophic bacteria (<0.8 μm) from phytoplankton and grazers and used unfiltered samples as controls (Fig. 1; Fig. S1). Filtration was performed using acid-washed filtration towers (ThermoScientific Nalgene) in an ISO Class 5 containerized clean room. We sampled the initial (unfiltered) and < 0.8 μm communities for community composition using flow cytometry and photosynthetic physiology using fast repetition rate fluorometry (FRRF) prior to incubation. Samples were then dispensed into 300 mL acid-washed polycarbonate bottles and spiked with 20 μCi of sodium ^14^C-bicarbonate and 0.2 nmol L^−1^ of an acidified ^55^Fe solution. Samples were incubated in temperature-controlled deckboard incubators at 10 ± 0.2°C under *in situ* irradiance (L) (12.5% incident irradiance = 19 m at SOTS at the time of sampling) and in the dark (D) to examine the dependency of Fe uptake and NPP on irradiance. An additional set of unfiltered samples were incubated in the light and in the presence of 1 μmol L^−1^ of the photosynthetic electron transport inhibitor 3-(3,4-dichlorophenyl)-1,1-dimethylurea (DCMU) (*N* = 3). After 24 hours incubations with ^14^C and ^55^Fe, the unfiltered and pre-incubation <0.8 μm filtration (Bact_PRE_) treatments were again size fractionated (post-incubation filtration) to separate the heterotrophic bacteria (0.2–0.8 μm) and phytoplankton communities (>0.8 μm) and the filters were rinsed with the Ti(III) EDTA— citrate reagent. Each treatment was performed in triplicate and sampled for flow cytometry, Fe uptake and NPP, and FRRF after 24 hours. A second set of triplicate samples were incubated without radionuclides for the first 24 hours, and then spiked with radionuclides at 24 hours and sampled at 48 hours.

Flow cytometry. Phytoplankton samples were fixed with formaldehyde-hexamine (18%:10% v/w) and bacterial samples were fixed with 2% glutaraldehyde (electron microscope grade, 25%). All flow cytometry samples were held at 4°C in the dark for 25–30 min after being fixed, then flash-frozen in liquid nitrogen and stored in a − 80°C freezer until analysis onshore [26]. The plankton community composition was determined with flow cytometry and divided into 8 groups: eukaryotes (Groups I-V), coccolithophores, picocyanobacteria, and heterotrophic bacteria. Eukaryotic phytoplankton were divided into five gates (I-V) based on violet laser excitation, red Chl a fluorescence, and the area-integrated forward scatter (FSC-A) signal as a proxy for phytoplankton size. Coccolithophores were identified by their unique side scatter profile. Picocyanobacteria were identified based on blue laser excitation and 581 nm emission. Bacterial counts were measured on the same instrument after staining the samples with SYBRG Green I (1000-fold dilution) and following a 15 min dark incubation period at room temperature. Gate positions were kept constant between treatments. The relative importance of each group in terms of population biovolume (F_pop_) was calculated using FSC-A converted to biovolume with a power regression. Biovolume was converted to C biomass using an equation for non-diatom phytoplankton [27]. Technical details of flow cytometry analyses are provided in SI Materials and Methods.

Fast repetition rate fluorometry (FRRF). FRRF was used to determine the maximum photochemical efficiency (F_v_/F_m_) of photosystem II (PSII) using a Light-induced Fluorescence Transients Fast Repetition Rate fluorometer (Soliense, Shoreham, NY, USA). After low light (2 μmol photons m^−2^ s^−1^) acclimation for approximately 30 min, samples were exposed to 140 flashes of light every 2.5 μsec (saturation sequence) to saturate PSII, after which the time interval between flashes was increased exponentially (relaxation sequence) for 90 flashes. F_v_/F_m_ (where F_v_ = F_m_ - F_o_) was calculated from F_o_ and F_m_, which refer to the minimum and maximum fluorescence, respectively, in the dark-acclimated state. F_v_/F_m_ was determined from the mean of 200 iterations of the fluorescence induction and relaxation protocol measured at 470 nm, and the average of at least 10 acquisitions is reported.

Statistical analyses We used linear mixed effect models to examine the effects of size fractions, light, and pre- versus post-filtration on the tested response variables using R (R “stats” packages) [28] as described previously [29] (Dataset S1). Technical details of the statistical analyses are provided in SI Materials and Methods.

## Results

**Figure 2:**
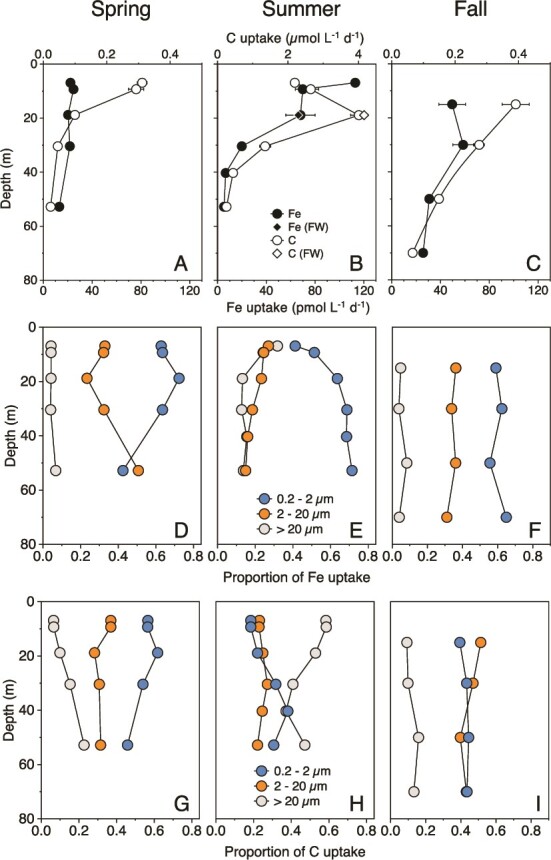
(A-C) Intracellular Fe and C uptake (i.e. NPP) rates measured in simulated depth profile incubations at SOTS during spring (October 2018), summer (December 2020), and fall (March 2018). The plotted data are the sum of the three size fractions. Open symbols = C uptake; closed symbols = Fe uptake. Diamonds show the results of the ferrous wheel (FW) experiment conducted on the same water sample that was collected from 19 m for the depth profile experiment in summer 2020. Note the different x-axis scale for C uptake in summer in panel B. Error bars are 1 s.d. (*N* = 2 except at 19 m for the summer profiles, where *N* = 3 for both the depth profile and ferrous wheel experiments). (D-F) Proportion of total Fe uptake by the three size fractions. (G-I) Proportion of total C uptake by each size fraction. Symbols for D-I: Blue = 0.2–2 μm; Orange = 2–20 μm; Gray = >20 μm.

*Seasonal Context*. The seasonal snapshots of Fe uptake and NPP at SOTS provide a broader context for the ferrous wheel manipulation experiments that we conducted in summer (Fig. 2). The average dissolved Fe concentrations in the mixed layer were 0.35 ± 0.03, 0.51 ± 0.02 and 0.32 ± 0.07 nmol L^−1^ in spring, summer, and fall, respectively (Table S1). Spring and fall are canonical HNLC conditions, with evidence for episodic Fe input from subtropical waters in summer [13] to further enhance the seasonal productivity maximum that characterizes HNLC regions [30]. Bioassay experiments show strong seasonal variation in phytoplankton Fe limitation at SOTS, with the greatest community response to Fe additions occurring in the summer despite the highest mixed layer dissolved Fe concentrations compared to those in the spring and fall [31].

We observed different-shaped vertical profiles in Fe uptake and NPP with season (Fig. 2). In the spring and fall, community Fe uptake rates at the greatest depth (i.e. lowest irradiance) were ~50% of surface rates, while community NPP was only 7%–17%. Trends in the depth profiles of both Fe uptake and NPP in the summer differed from those in the spring and fall. We observed higher rates of NPP and Fe uptake and a subsurface peak in NPP in summer. Both Fe uptake and NPP declined to a similar extent with depth (5 and 12% of surface values, respectively) such that the decoupling of Fe and NPP with depth observed in other seasons was not apparent. Flow cytometry revealed that the <2 μm phytoplankton were picoeukaryotes (>92% of <2 μm phytoplankton biomass). In contrast, cyanobacteria were very low in abundance (<8% of <2 μm phytoplankton biomass; 1% of total phytoplankton biomass; Table S2). Visual microscopy revealed that the >20 μm fraction was dominated by diatoms and dinoflagellates (Fig. S2). Heterotrophic bacteria accounted for 51% of the community biomass (Table S2).

For each season, and for most euphotic zone depths, Fe uptake was dominated by the <2 μm fraction, comprising ~60% of the total pelagic uptake (Fig. 2). In contrast, Fe uptake was lowest in the >20 μm fraction (5–25%). Small cells also dominated NPP in spring and fall; the ~10-fold increase in NPP in the summer was principally due to >20 μm cells, which contributed ~56% to column-integrated NPP. This changed the community contribution to Fe uptake and NPP, and the shape of the summer depth profile relative to those in spring and fall. The decoupling of Fe and C uptake (i.e. NPP) rates in the spring and fall resulted in very high Fe:C uptake ratios, particularly at the base of the euphotic zone (spring ~600 μmol:mol; fall ~400 μmol:mol; Fig. S3).

**Figure 3:**
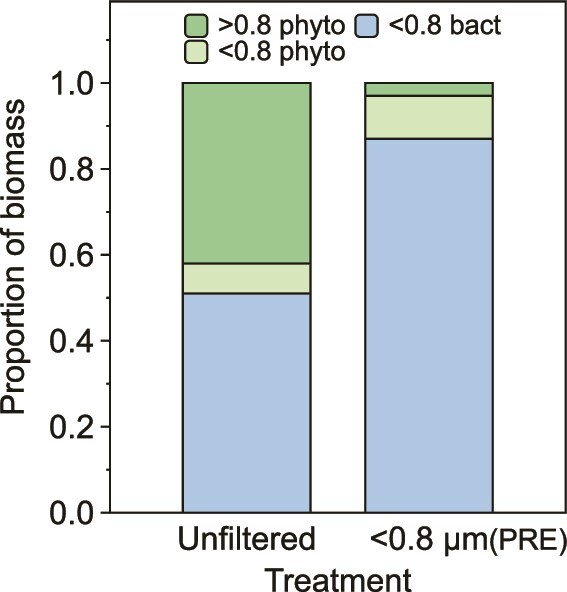
The initial (pre-incubation) distributions of biomass determined using flow cytometry in the unfiltered and the PRE-incubation <0.8 μm filtered (PRE) treatments of the ferrous wheel experiment conducted in summer 2020 at SOTS. The <0.8 μm fraction was further broken down into heterotrophic bacteria (<0.8 bacteria; blue) and phytoplankton (<0.8 phyto; light green).

*Effective size separation of phytoplankton and bacteria*. Methods to separate microbial groups include antibiotics [32], but we sought a less invasive technique for the ferrous wheel experiment. A distinct size cut-off of 0.8 μm between heterotrophic bacteria and picophytoplankton has been reported using atomic force microscopy [33]. In our experiments, filtration through a 0.8 μm porosity filter effectively separated phytoplankton from heterotrophic bacteria (Fig. 3; Table S2). Chlorophyll fluorescence confirmed the flow cytometry results (92–95% of phytoplankton were retained by a 0.8 μm filter; Fig. S4). Hereafter, we operationally define the <0.8 μm fraction as “heterotrophic bacteria” and the >0.8 μm fraction as “phytoplankton”. Photosynthetic efficiency was constant in both the light and dark unfiltered treatments (~0.5) and was not detected in the <0.8 μm pre-incubation filtered (Bact_PRE_) treatments (Fig. S4). The distribution of biovolume between photoautotrophs and heterotrophic bacteria did not significantly change during the 48 hours incubation, and the very low vacuum used to filter the samples (<10 mm Hg) did not appear to damage the cells (Fig. S5).

The ferrous wheel experiment was conducted in summer 2020 using seawater from 19 m depth (i.e. the subsurface NPP maximum). Samples were collected from the same sampling bottle as the 24 hours simulated *in situ* depth profile incubation (Fig. S1) and the Fe uptake and NPP rates of the parallel incubations were comparable (Fig. 2B; Table S3). We computed the 0.8–2.0 μm contribution to Fe and C uptake by subtracting the 0.2–0.8 μm fraction of the unfiltered treatment of the ferrous wheel experiment from the 0.2–2 μm fraction of the depth profile experiment. This enabled us to further dissect the <2 μm fraction into heterotrophic bacteria (0.2–0.8 μm) and picoeukaryotic phytoplankton (0.8–2.0 μm) contributions to Fe uptake and NPP.

**Figure 4:**
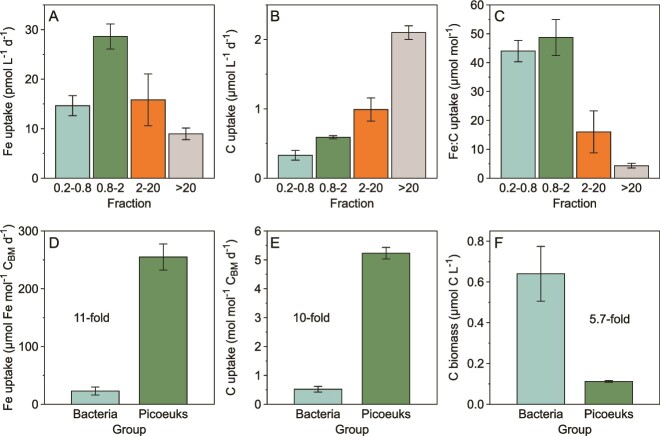
Size-fractionated rates of Fe uptake (A), inorganic C uptake (i.e. NPP) (B) and the Fe:C uptake ratio (C) determined by combining the results of the ferrous wheel and depth profile experiments. Carbon biomass (C_BM_) was calculated for the size-fractionated populations that could be comprehensively enumerated using flow cytometry (F). Iron (D) and carbon (E) uptake rates were normalized to C_BM_ for these populations (heterotrophic bacteria (“bacteria”; 0.2–0.8 μm) and picoeukaryotic phytoplankton (“Picoeuks”; 0.8–2 μm)). Panel E is equivalent to the C turnover times for the heterotrophic bacteria (~0.5 d^−1^) and picoeukaryotic phytoplankton (5 d^−1^). Care must be taken when interpreting Fe:C *uptake* ratios, as Fe uptake rates may not reflect steady-state Fe demand, and inorganic C uptake largely reflects phototrophic uptake over the 24–48 hours timescale of our experiments. The error bars are 1 s.d. (*N* = 3).

*Picoeukaryotic phytoplankton dominate Fe uptake.* By partitioning Fe uptake between the bacterial and 3 phytoplankton fractions we calculated that heterotrophic bacteria accounted for 21% of total Fe uptake, while phytoplankton accounted for 79% (Fig. 4A). Contrary to the notion that heterotrophic bacteria dominate Fe uptake in the <2 μm fraction [13, 16], the picoeukaryotic phytoplankton fraction (0.8–2 μm) took up Fe ~2-fold faster than heterotrophic bacteria (*P* < 0.05, Dataset S1.2) and accounted for 42% of community Fe uptake. As shown previously in Fig. 2, the >2 μm phytoplankton took up Fe at lower rates (Fig. 4A). In contrast, the >2 μm fraction dominated NPP (77%, Fig. 4B; *P* < 0.05, Dataset S1.3). Together, these trends manifested as comparable Fe:C uptake ratios for heterotrophic bacteria and picoeukaryotic phytoplankton, which were 4- to 8-fold greater than those for phytoplankton in the 2–20 and > 20 μm fractions (Fig. 4C; *P* < 0.05, Dataset S1.4). Despite the higher throughput of Fe and C in picoeukaryotes, these elements scale proportionately. Consequently, the Fe:C uptake ratio of heterotrophic bacteria and picoeukaryotes was ~45 μmol:mol (Fig. 4C).

We enumerated the <2 μm fraction using flow cytometry, enabling calculation of C biomass for the heterotrophic bacteria and picoeukaryotes (Table S4). We then normalized their Fe and C uptake rates to C biomass (Fig. 4A,B). After normalization, the rate of Fe uptake by picoeukaryotes was 10-fold faster than for heterotrophic bacteria (Fig. 4D; *P* < 0.05, Dataset S1.5). This was also the case for NPP normalized to C biomass (Fig. 4E; *P* < 0.05, Dataset S1.6). Returning to Fig. 4A, the 2-fold greater Fe uptake by picoeukaryotes than by heterotrophic bacteria reflects a combination of 10-fold greater C biomass-normalized Fe uptake rates in picoeukaryotes, which is partially compensated for by an ~6-fold greater heterotrophic bacteria C biomass (Fig. 4F).

**Figure 5:**
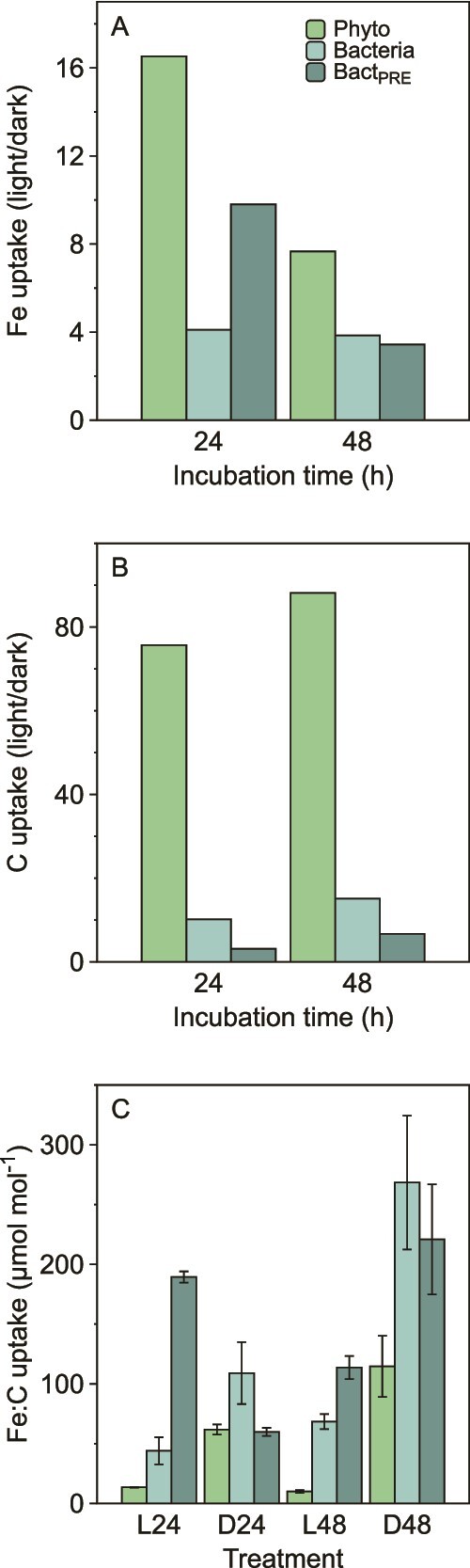
The difference in uptake rates under *in situ* incident irradiance (12.5% of incident irradiance corresponding to the 19 m depth of collection) compared to that in the dark (light/dark). (A) Fe uptake. (B) Inorganic C uptake. (C) the Fe:C uptake ratio in phytoplankton (Phyto), and heterotrophic bacteria in the presence (bacteria) or absence (Bact_PRE_) of phytoplankton in the light (L) and dark (D) after 24 (L24, D24) and 48 hours (L48, D48) of incubation. The error bars are 1 s.d. (*N* = 3).

*Light accelerates Fe uptake in both phytoplankton and heterotrophic bacteria.* We routinely observe seasonal differences in the shape of the depth profiles of Fe uptake and NPP at SOTS (Fig. 2), with implications for the coupling of the Fe and C cycles in the euphotic zone. Our experimental design allowed us to explore these trends further (Fig. 5). We incubated samples under ambient irradiance (12.5% incident irradiance; 83 μmol photons m^−2^ s^−1^ average daily PAR) and in the dark to probe the dependence of Fe uptake and NPP on irradiance within the phytoplankton and heterotrophic bacteria. We repeated the experiment twice at 24 hours intervals. We also compared the results between the unfiltered and the pre-incubation fractionated treatment (Fig. 1; Fig. S1). We observed an 8- to 16-fold increase in Fe uptake by phytoplankton in the light versus the dark (Fig. 5; Fig. S6). Light also increased Fe uptake in heterotrophic bacteria by 4- to 8-fold. In the treatment in which heterotrophic bacteria were isolated from the ferrous wheel (i.e. Bact_PRE_; Fig. 1), we observed an 8-fold increase in Fe uptake after 24 hours (*c.f.* 2-fold increase over the bacteria not isolated from the ferrous wheel). These light-dependent trends in Fe uptake were more muted after 48 hours (Dataset S1.7).

For NPP, the light effect on phytoplankton was ~80-fold, reflecting the obligate need for light for photosynthesis. We observed a 10-fold increase in NPP in the <0.8 μm fraction, potentially due to the small but highly influential contribution of phytoplankton that passed through the 0.8 μm filter (~15% of the fraction biovolume (F_pop_), ~8% of the fraction C biomass; Fig. 3; Table S2; Fig. S7). However, for the Bact_PRE_ treatment there was only a 3-fold increase in NPP in the light (Fig. 5B).

We observed a 200-fold range in Fe:C uptake ratios across the different microbial groups and in the light vs. the dark (Fig. 5C). Despite irradiance increasing Fe uptake, the effect on C uptake was still greater. Consequently, when considering the entire microbial community together, Fe:C uptake ratios were greater in the dark and for smaller microbes (Fig. 4C). Therefore, these wide-ranging Fe:C uptake ratios reflect both community composition and environmental (in this case, light) forcing. Importantly, in the context of the seasonal cycle at SOTS, we performed this experiment in the summer, when the decoupling of Fe and C uptake was the least of the three seasons studied (Fig. 2A-C; Fig. S3).

## Discussion

Small cells dominate Fe uptake and are therefore key players in the ferrous wheel (Fig. 2). Our experiment isolated the heterotrophic bacteria from picophytoplankton that are similarly sized but primarily differ in trophic mode and Fe acquisition strategies [34–36]. Heterotrophic bacteria have significant Fe requirements, possibly exceeding those of phytoplankton [37–39]. It has been hypothesized that bacteria are superior competitors for nutrient acquisition based on their small size and high surface area:volume (SA:V) ratio [22, 37]. In field studies, it has been further assumed that the majority of Fe uptake in the <2 μm fraction is by bacteria [13, 16]. Our findings challenge this notion and provide insights into the drivers of Fe uptake by both picoeukaryotic phytoplankton and heterotrophic bacteria.

Overall, in the ferrous wheel experiment phytoplankton took up ~79% of Fe, higher than the partitioning observed previously (e.g. 60–70% in the Northeast Pacific [14, 37]; 50% at SOTS in fall [15]). Our most arresting result was that picoeukaryotes take up the majority of Fe. They accounted for 53% of phytoplankton Fe uptake, and 42% of total Fe uptake (phytoplankton + bacteria). In contrast, heterotrophic bacteria accounted for only 21% of total Fe uptake. Picoeukaryotes are comparable to heterotrophic bacteria in size, and hence in their SA:V ratios (Table S4). But, when normalized to C biomass, the Fe uptake rates of the slightly larger picoeukaryotes were 10-fold greater than those of heterotrophic bacteria (Fig. 4D). Our finding that the Fe uptake rates of picoeukaryotes greatly exceed those of heterotrophic bacteria challenges the notion that bacteria require more Fe per unit of C biomass [16].

To provide context for the higher Fe uptake rates in picoeukaryotes compared to heterotrophic bacteria observed in our study, we calculated the surface area-normalized dissolved Fe uptake rate constant, k_in-app_ /SA [40] (Table S5). Picoeukaryotes had a k_in-app_/SA 6-fold higher than the global mean (of 560 single cell *k*_in-app_/SA values from 31 low-Fe stations) [41]. In marked contrast, the k_in-app_/SA of heterotrophic bacteria was 6-fold lower than the global mean. We calculated a similarly low k_in-app_/SA for Fe-limited heterotrophic bacteria at SOTS in the fall [15]. These calculations not only challenge the notion that heterotrophic bacteria are superior competitors for Fe acquisition but also reveal an unexpectedly effective Fe acquisition apparatus in picoeukaryotes.

Small cells can dominate photosynthetic biomass and primary production in many marine ecosystems [42] including the Southern Ocean [43]. Who are the Southern Ocean picoeukaryotes? In short, we do not know. Their small size and lack of morphological features hinders traditional taxonomic identification [44]. Indeed, they are referred to as URGOs (Unidentified Round Green Objects) [44] or UNANs (Unidentified NANoflagellates) [45]. They are most likely haptophytes, prasinophytes, and chlorophytes based on HPLC pigment analyses [45]. Improved tools, such as more and better annotations of marine eukaryotic genomes, are needed to better characterize the taxonomy and ecological functions of these organisms. An open question arising from our study is whether the fate of picoeukaryotes within the ferrous wheel differs from that of heterotrophic bacteria. The most sophisticated biogeochemical models (e.g. PISCES-NEMO) represent diatoms, nanoflagellates, and heterotrophic bacteria but not this potentially important picoeukaryote group [12, 46].

Light influences several processes that affect both Fe and C biogeochemistry (Fig. 1). The simulated *in situ* depth-profile incubations revealed less change in Fe uptake than in NPP in the <2 μm fraction, particularly in the spring and fall (Fig. 2). We assumed that this trend was due to heterotrophic bacteria that take up Fe but negligible amounts of inorganic C. What we observed was more nuanced. As expected, light had a greater influence on the NPP of photosynthetic phytoplankton (>0.8 μm) than on that of heterotrophic bacteria. However, what was unexpected was the major influence that light had on Fe uptake by both phytoplankton and heterotrophic bacteria; an ~12-fold stimulation of phytoplankton (>0.8 μm), but also a ~ 5-fold stimulation of the heterotrophic bacteria (0.2–0.8 μm; ~85% bacterial biovolume (F_pop_), ~92% bacterial C biomass). Our study also shows that this light stimulation of Fe uptake occurs for heterotrophic bacteria in the absence of a “new” source of DOC (i.e. potentially a source of natural ligands) from phytoplankton (Fig. 5) and in the presence of the photosynthetic electron transport inhibitor DCMU (Fig. S7).

A 15-fold light stimulation of Fe uptake from *in situ* ligands in a cyanobacteria-dominated subantarctic community has been reported previously [18], a result that has been observed elsewhere [40, 47]. In contrast, only a 2-fold light stimulation of Fe uptake is evident when using photolabile “model” ligands [18, 40, 47]. These observations suggest that natural *in situ* ligands have unknown photochemical properties and interactions that are not captured well by model ligands with respect to stimulating Fe uptake. However, there are some compelling clues in the literature to explain these observations. Vibrioferrin, a bacterially produced Fe-binding ligand (i.e. a siderophore) is photochemically reactive and increases Fe uptake in both phytoplankton and heterotrophic bacteria but differentially (>20-fold vs 1.7-fold, respectively) [20]. This mutualistic relationship is hypothesized to be effective within the diffusive boundary layer (i.e. the phycosphere) of larger cells. The effectiveness of this bacterial–algal mutualism in picoeukaryotes is unclear because as the phycosphere decreases with cell size it becomes indistinguishable from surrounding seawater [48]. Alternatively, a pool of weak but abundant ligands such as polysaccharides (a constituent of the labile DOC pool) may facilitate Fe retention and uptake if its size increases in the light via increased exudation by phytoplankton [49].

In addition to photochemistry, light may stimulate microbial Fe uptake at the physiological level. Light induces a physiological response that increases Fe uptake in marine diatoms [50]. However, high-light grown diatoms retain their enhanced ability to take up Fe in the dark (for at least 4 h) [50], which would likely mitigate the differences in light vs dark Fe uptake by phytoplankton that we observed (Fig. 5; Fig. S6). Photoheterotrophy is a widespread mode of bacterial metabolism, notably in the oligotrophic surface ocean, where microbes experience chronic nutrient limitation. One especially widespread form of photoheterotrophy is based on proteorhodopsin, which uses light to generate proton motive force that can drive ATP synthesis or nutrient uptake [51]. However, the role of proteorhodopsin in bacterial Fe metabolism is unclear [52]. To date, there are no reports on the physiological effect of light on bacterial Fe uptake. Cumulatively, these results shed light on how little is known about the environmental drivers of Fe uptake, the identity of Fe-binding ligands, and the effects of ligands on Fe bioavailability. There is no smoking gun; it is likely that a combination of mechanisms contributes to the light stimulation of Fe uptake that we observed.

Pre-incubation size fractionation isolated heterotrophic bacteria from the ferrous wheel (Fig. 1). Comparison with post-incubation size fractionation allowed us to potentially demarcate the effects of isolating heterotrophic bacteria from the ferrous wheel and the effect of light on Fe uptake by both phytoplankton and heterotrophic bacteria. We found that in the absence of phytoplankton, higher Fe uptake rates by bacteria were observed in the light after 24 hours incubation but not after 48 hours (Fig. 5; Fig. S6). Isolating bacteria not only removed competition with phytoplankton for Fe but also shut off the immediate source of labile DOC (i.e. algal exudation), which may play dual roles as a C source and a pool of Fe-binding ligands. Our results suggest that altering both factors (DOC supply and Fe competition) was a zero-sum game under our experimental conditions. The transient increase we observed could be due to the presence of a residual labile DOC pool and/or a reduction in competition for Fe. After 48 hours the light-modulated advantage of bacteria isolated from the ferrous wheel disappeared, and there was no difference in Fe uptake rates by bacteria in the presence or absence of phytoplankton. Our findings point to tight coupling across both Fe and C and different trophic levels in the microbially mediated ferrous wheel.

## Conclusions

Prior comparisons of the relative contributions of recycled and new Fe have focused on the Fe-ratio [7, 9]. However, the rate of recycling over the daily light:dark cycle has never been factored into the relative contributions of new and regenerated Fe. Our findings show that rates of Fe uptake, and presumably then recycling, differ between picoeukaryotic phytoplankton and heterotrophic bacteria: picoeukaryotes are a small biogenic Fe pool that spins very quickly compared to the larger but slower turning Fe pool of heterotrophic bacteria. This finding – of two different ferrous cogs – has potential, but unknown, implications for the rate and possibly fates of recycled Fe [8, 9]. Furthermore, our findings suggest that light can increase the rate at which Fe is shuttled through the ferrous wheel relative to that in the dark. The diel expression of microbial Fe transporter transcripts reinforces the idea of diurnal patterns in the supply term of the ferrous wheel [53]. In addition to the diurnal cycle, the influence of these light-mediated processes has wider implications for rates of Fe recycling with season.

The trends reported in Fig. 2 capture both changes in ambient light over the season and the relationship between the depth of the euphotic zone. As such they place our summertime measurements (an upper bound for Fe uptake over the season) in a wider context and point to the need for additional metrics. Specifically, we need seasonal Fe uptake versus irradiance curves for heterotrophic bacteria and phytoplankton (normalized to their respective biomasses). To determine the relationship between Fe and C biogeochemistry, we also need complimentary measurements of NPP.

Our summertime findings suggest that prior observations of relative constancy in Fe uptake with depth [13] are not applicable across the annual cycle. We estimated that irradiance in the mixed layer was 4.5- and 14-fold greater in the summer compared to the spring and fall, respectively (Table S1). Is there a threshold in mean water column irradiance that triggers a transition in Fe uptake for the phytoplankton, and is the increase linked to the 10-fold increase in NPP? Furthermore, how are these changes in phytoplankton NPP and Fe uptake influenced by mixotrophy and how do they influence the daily and seasonal bacterial demands for Fe and C [15, 54–56]? While our summertime findings of light-mediated increases in Fe uptake by phytoplankton are not surprising [18, 40], our new observation of light-stimulated Fe uptake in heterotrophic bacteria raises questions about the underlying mechanism(s).

Taken together our findings point to future research priorities that focus on how the Fe uptake components of the ferrous wheel function across the diel cycle. If as we propose that light can increase the rate at which Fe is shuttled through the ferrous wheel relative to that in the dark and does so differently for heterotrophic bacteria compared to phytoplankton, then there may be a fundamental shift in the balance of Fe uptake vs recycling over the diel cycle. This will be superimposed on cycles of particle production and destruction driven by other ecological processes [57]. Understanding this superimposition will require the intersection of ferrous wheel dynamics with the known sequence of processes within the microbial loop over the diurnal cycle [58, 59].

## Supplementary Material

Strzepek_ISME_SI_R4_wraf015

Strzepek_ISME_DatasetS1_wraf015

## Data Availability

The iron and carbon uptake datasets generated during the current study are available at the IMAS Metadata Catalogue https://doi.org/10.25959/9G97-VH65.
