# Supplementary material for: Microbial competition for iron determines its availability to the ferrous wheel
Source: ISME J. 2025 Jan 27;19(1):wraf015. doi: 10.1093/ismejo/wraf015 (PMC11833320; doi:10.1093/ismejo/wraf015)
Supplement: Strzepek_ISME_SI_R4_wraf015 [file strzepek_isme_si_r4_wraf015.docx]

**Supplementary Information for**

**Microbial competition for iron determines its availability to the Ferrous Wheel**

Robert F. Strzepek^1^*, Pauline Latour^2,3^, Michael J. Ellwood^4^, Yeala Shaked^5,6^, Philip W. Boyd^1,3^

^1^ Australian Antarctic Program Partnership (AAPP), Institute for Marine and Antarctic Studies, University of Tasmania, 20 Castray Esplanade, Battery Point, TAS, 7004, Australia

^2^ ARC Australian Centre for Excellence in Antarctic Sciences (ACEAS), University of Tasmania, Hobart, TAS, Australia.

^3^ Institute for Marine and Antarctic Studies (IMAS), University of Tasmania, Hobart, TAS, Australia

^4^ Research School of Earth Sciences, Australian National University, Acton, ACT, Australia

^5^ The Fredy and Nadine Herrmann Institute of Earth Sciences, Hebrew University of Jerusalem, Jerusalem, Israel

^6^ The Interuniversity Institute for Marine Sciences in Eilat, Eilat, Israel

***Corresponding author:** Robert F. Strzepek

**Email:** robert.strzepek@utas.edu.au

**This PDF file includes:**

Figures S1 to S7

Tables S1 to S5

SI Materials and Methods

SI References

**Supplementary Information**

Fig. S1. Experimental design of the ferrous wheel and simulated *in situ* depth profile experiments. All post-incubation filtrations included a wash with the Ti(III)-EDTA-citrate reagent to remove extracellularly-bound Fe. *The depth profiles at 19 m in summer were conducted in triplicate. All other intracellular, size-fractionated depth profile filtrations were conducted in duplicate and had one “dark correction” per depth. ^†^An additional set of unfiltered samples were incubated in the light and in the presence of 1 µmol L^-1^ of the photosynthetic electron transport inhibitor 3-(3,4-dichlorophenyl)-1,1-dimethylurea (DCMU) during the first 24 h incubation (*N*=3).


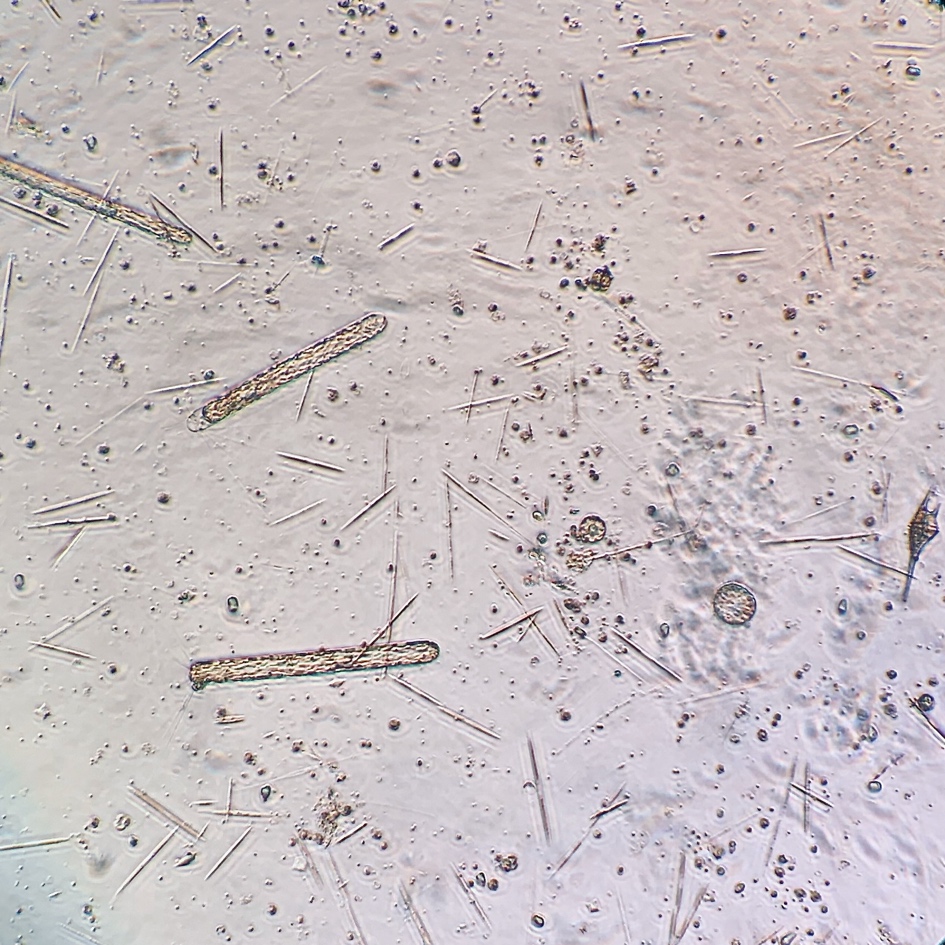


Fig. S2. Photomicrograph of a 20-fold concentrate of the phytoplankton community from 19 m depth at the Southern Ocean Time Series (SOTS) site in summer 2020. The >20 µm fraction was principally composed of diatoms (e.g. *Corethron* sp., *Pseudo-nitzschia* sp., and *Cylindrotheca* sp.,) and dinoflagellates (e.g. *Tripos* sp.).

Fig. S3. Intracellular Fe and C uptake rates. Size-fractionated profiles of Fe (A-C), and carbon (D-F) uptake and the Fe:C uptake ratio (G-I) versus depth for spring (A, D, G), summer (B, E, H) and fall (C, F, I) at SOTS. Error bars are1 s.d. (*N*=2 except at 19 m depth in summer, where *N*=3).

Fig. S4. (A) Chlorophyll fluorescence (F_o_) measured using FRRF in the unfiltered compared to the 0.8 µm pre-incubation filtered (Bact_PRE_) treatments immediately after pre-incubation size fractionation (T0) and after 24h and 48h of incubation in the light (L24, L48) and dark (D24, D48) treatments. A total of 92-98% of the total chlorophyll fluorescence was measured in the >0.8 µm fraction. (B) F_v_/F_m_ in the unfiltered treatments. The chlorophyll fluorescence signal was too low to accurately measure F_v_/F_m_ in the Bact_PRE_ treatments. Error bars are 1 s.d. (*N*=3).

Fig. S5. Community composition of the ferrous wheel experiment over the 48h duration of the experiment. Community composition is presented as biovolume by multiplying the biovolume (µm^3^) by the count per µL of sample within each of the gated populations. Biovolume was converted from area-integrated forward scatter (*FSC-A*) measured by flow cytometry using a power regression. Populations are presented in descending order of cell size. (A) Unfiltered treatment in the light (12.5% incident irradiance). (B) Unfiltered treatment in the dark. (C) 0.8 µm pre-incubation filtered treatment (Bact_PRE_) in the light. (D) 0.8 µm pre-incubation filtered treatment (Bact_PRE_) in the dark.

Fig. S6. Iron uptake rates of phytoplankton (A) and heterotrophic bacteria (B) in the ferrous wheel experiment. For the heterotrophic bacteria, we compared populations isolated from phytoplankton by pre-incubation size fractionation (Pre) with bacteria incubated with phytoplankton and then isolated by post-incubation size fractionation (Post). Incubations were conducted in the light and the dark and sampled after 24h (L24, D24) and 48h (L48, D48). (C) Iron uptake rates of bacteria normalized to bacterial C biomass. Error bars are 1 s.d. (*N*=3).

Fig. S7. The effect of light, dark, and light plus the photosynthetic electron transport inhibitor DCMU on (A) Fe and (B) inorganic C uptake in the >0.8 µm (phytoplankton) and 0.2-0.8 µm (heterotrophic bacteria) fractions in 24 h incubations. Error bars are 1 s.d. (*N*=3).

Table S1. Mixed layer depths, surface PAR, light attenuation coefficients, and dissolved Fe concentrations in the spring, summer, and fall at SOTS.

| Season | Experiment  Date | MLD^a^  (m) | Surface PAR^b^ | K  (m^-1^)^c^ | PAR Index^d^ | dFe  (nmol L^-1^)^e^ |
| --- | --- | --- | --- | --- | --- | --- |
| Spring | 2 Oct 2018 | 120 | 385 | 0.04 | 73 | 0.35 ± 0.03 |
| Summer | 18 Dec 2020 | 19 | 698 | 0.11 | 330 | 0.51 ± 0.02 |
| Fall | 18 Mar 2018 | 95 | 244 | 0.11 | 24 | 0.32 ± 0.07 |

^a^ Mixed layer depths (MLD) were computed following Holte and Talley (2009) [1].

^b^ Water surface photosynthetically active radiation (PAR). Units: µmol photons m^-2^ s^-1^. 8-day 4 km area-averaged time series from MODIS-Aqua (https://giovanni.gsfc.nasa.gov).

^c^ Light attenuation coefficient. K = (D(ln(PAR)))/(D(z)), where D(ln(PAR)) is the difference between the ln(PAR) measured between two depths and D(z) the difference between the two depths. The PAR data was obtained from midday bio-optics CTD casts.

^d^ Synthetic PAR index calculated as: (Surface PAR/MLD)/K.

^e^ Mean mixed-layer dissolved Fe concentrations (dFe) ± 1 s.d. (*N*=2-4). For the calculation of K_in-app_/SA in the summer ferrous wheel experiment, the 0.2 nmol L^-1^ of ^55^Fe used to measure Fe uptake was added to the summer dFe value.

Table S2. Contribution of plankton groups to phytoplankton and community (phytoplankton + heterotrophic bacteria) biomass in the unfiltered and <0.8 µm pre-filtered (Bact_PRE_) treatments. Biomass was calculated from flow cytometry data as F_pop_, the relative importance of each group in terms of their biovolume and abundance.

| Fraction (µm) | Group | Phytoplankton biomass (%) | | |  | Total biomass  (%) | |
| --- | --- | --- | --- | --- | --- | --- | --- |
|  |  | Unfiltered | <0.8 µm |  | Unfiltered | | <0.8 µm |
| 0.2–0.8 | Heterotrophic bacteria | / | / |  | 51.2 | | 86.2 |
|  | Picocyanobacteria | 1.2 | 3.0 |  | 0.6 | | 0.4 |
|  | Eukaryote1 | 6.5 | 42.5 |  | 3.2 | | 5.9 |
| 0.8-2 | Eukaryote2 | 6.6 | 16.6 |  | 3.2 | | 2.3 |
|  | Eukaryote3 | 24.9 | 10.4 |  | 12.2 | | 1.4 |
|  | Eukaryote4 | 21.7 | 11.1 |  | 10.6 | | 1.5 |
| >2 | Coccolithophores | 9.3 | 4.3 |  | 4.5 | | 0.6 |
|  | Eukaryote5 | 29.8 | 12.1 |  | 14.5 | | 1.7 |

**Table S3.** Comparison of Fe and C uptake rates from a simulated *in situ* deckboard experiment (i.e. depth profile) and the ferrous wheel experiment performed in parallel using water collected from 19 m at SOTS in the summer. Errors are 1 s.d. (*N*=3).

| Experiment | Fraction (µm) | Fe uptake  (pmol L^-1^ d^-1^) | C uptake  (µmol L^-1^ d^-1^) | Fe:C  (µmol:mol) |
| --- | --- | --- | --- | --- |
| Ferrous wheel | 0.2-0.8 | 14.6 ± 2.0 | 0.33 ± 0.07 | 44 ± 4 |
|  | >0.8 | 51.6 ± 3.0 | 3.83 ± 0.02 | 14 ± 1 |
|  | Sum of fractions | **66.2 ± 4.8** | **4.16 ± 0.10** |  |
| Depth profile | 0.2-2 | 43.3 ± 1.5 | 0.92 ± 0.05 | 47 ± 15 |
|  | 2-20 | 15.8 ± 5.2 | 0.99 ± 0.17 | 16 ± 7 |
|  | >20 | 9.0 ± 1.2 | 2.10 ± 0.10 | 4.3 ± 0.8 |
|  | Sum of fractions | **68.0 ± 12** | **4.01 ± 0.23** |  |
| Combined^a^ | 0.2-0.8 | 14.6 ± 2.0 | 0.33 ± 0.07 | 44 ± 4 |
|  | 0.8-2 | 28.6 ± 2.5 | 0.59 ± 0.02 | 49 ± 6 |
|  | 2-20 | 15.8 ± 5.2 | 0.99 ± 0.17 | 16 ± 7 |
|  | >20 | 9.0 ± 1.2 | 2.10 ± 0.10 | 4.3 ± 0.8 |

^a^ The 0.2-0.8 µm fraction of the ferrous wheel experiment was subtracted from the 0.2-2 µm fraction of the depth profile experiment to compute the 0.8-2.0 µm contribution to Fe and C uptake.

**Table S4.** Estimates of cell volume (biovolume) for plankton groups using area-integrated forward scatter (*FSC-A*). Cell diameter and surface area were calculated assuming that the cells were of spherical geometry. Biovolume was converted to carbon per unit of cell volume using the non-diatom equation of Menden-Deuer and Lessar (2000) [2].

| Fraction (µm) | Group | Biovolume  (µm^3^) | Diameter  (µm)^a^ | SA:V  (µm^-1^) | Carbon  (fg µm^-3^)^b^ |
| --- | --- | --- | --- | --- | --- |
|  |  |  |  |  |  |
| 0.2–0.8 | Heterotrophic bacteria | 0.018 ± 0.002 | 0.32 | 19 | 276 |
|  | Picocyanobacteria | 0.029 ± 0.008 | 0.38 | 16 | 204 |
|  | Eukaryote1 | 0.083 ± 0.030 | 0.54 | 11 | 251 |
| 0.8-2 | Eukaryote2 | 0.36 ±0.11 | 0.88 | 6.8 | 229 |
|  | Eukaryote3 | 1.6 ± 0.14 | 1.5 | 4.1 | 210 |
|  | Eukaryote4 | 5.3 ± 1.1 | 2.2 | 2.8 | 195 |
| >2 | Coccolithophores | 9.3 ± 2.4 | 2.6 | 2.3 | 188 |
|  | Eukaryote5 | 19 ± 4.3 | 3.3 | 1.8 | 180 |

^a^ The Eukaryote 2 group straddled the 0.8 µm cutoff for the size-fractionated treatment. The contribution of this group to both the unfiltered and <0.8 µm fractions was calculated by difference. The fact that this group, with an estimated cell diameter of 0.88 µm, straddles this 0.8 µm cutoff provides independent evidence that the calculated biovolumes and our assumption of spherical geometry are reasonable.

^b^ Our carbon estimate for heterotrophic bacteria equates to 4.9 fg cell^-1^, within the 2.9–6.5 fg cell^-1^ range reported for bacteria from oligotrophic regions [3, 4]. Similarly, our C estimate for picoeukaryotes (221 ± 24 fg µm^-3^; mean of Eukaryote groups 1-4) compares favorably to published values of 220 fg µm^-3^ [5] and 200 fg µm^-3^ [6].

**Table S5.** Surface area-normalized dissolved Fe uptake rate constants, k_in-app_/SA, for picoeukaryotes and heterotrophic bacteria at SOTS.

| Group | k_in-app_/SA (L μm^-2^ d^-1^)^a^ | Study |
| --- | --- | --- |
| Picoeukaryotes (0.8-2 µm) | 2.3 ± 1.0 x 10^-9^ | This study^b^ |
| Heterotrophic bacteria (0.2-0.8 µm) | 4.8 ± 1.2 x 10^-11^ | This study^b^ |
| Heterotrophic bacteria (0.2-0.8 µm) | 6.5 ± 2.9 x 10^-11^ | Fourquez et al., (2020)^c^ |
| Global Mean | 3.2 ± 0.8 × 10^−10^ | Shaked et al., (2021)^d^ |

^a^ k_in-app_/SA was computed by dividing Fe uptake rates by the total dFe concentration (0.50 nmol L^-1^ *in situ* at 20 m + 0.2 nmol L^-1^ ^55^Fe addition) normalized to cell surface area (SA) (*viz*. k_in-app_/SA = (Fe uptake/dFe)/SA).

^b^ Average daily PAR = 83 µmol photons m^− 2^ s^− 1^; 10 ± 0.2°C; Summer

^c^ in controls (no additions); assuming bacteria diameter = 0.6 µm; average daily PAR = 4.5 μmol photons m^−2^ s^−1^; 13.5°C; fall; [7]

^d^ dim light; *in situ* 20 m; [8]

SI Materials and Methods.

Technical details for “Flow cytometry”. Samples were analysed at the Menzies Institute for Medical Research (University of Tasmania, Hobart) using an Aurora Cytek flow cytometer (Cytek Biosciences, Fremont, CA, USA). Frozen samples were thawed at 37 °C for 5–10 min before running 500 µL of unstained samples at flow rates of approximately 50 µL min^–1^, using Milli-Q water as sheath fluid. Violet and blue excitation lights were used to differentiate the main phytoplankton groups through their fluorescence pigments: chlorophyll with red fluorescence and phycoerythrin with orange fluorescence, respectively, against forward scatter (FSC). All scatter and fluorescence parameters were analysed based on values from the integrated area of the excitation peak. Results obtained were analysed using SpectroFlo software (Cytek Biosciences). Eukaryotic phytoplankton communities were divided into five gates: (I-V), identified on the violet channel (V12, 405 nm excitation, 692 nm emission) against FSC. If the signal from V12 was saturated, we used another excitation wavelength (B7, 488 nm excitation, 661 nm emission). Coccolithophores were identified by their side scatter profile. Picocyanobacteria were identified on another fluorescence channel (B4, 488 nm excitation, 581 nm emission) due to the presence of phycoerythrin [9]. Cell counts per unit volume were determined from the instrument through the known volume analysed. Heterotrophic bacterial counts were performed on thawed fixed samples after the addition of SYBR Green I stain (1000-fold dilution). Samples were incubated with the stain for 15 min at room temperature in the dark. Then, a 50 µL aliquot of stained sample was run on the instrument at a high flow rate. Bacteria were identified using blue excitation and green fluorescence (B2, 488 nm excitation, 525 nm emission).

We then used the cell counts to calculate the relative importance of each group by comparing their biovolume and abundance, using the following equation from Bach et al., (2018) [10]:

$\boldsymbol{F}_{\boldsymbol{pop}}\boldsymbol{=}\frac{\boldsymbol{N}_{\boldsymbol{pop}}\boldsymbol{\times}\boldsymbol{Biovolume}_{\boldsymbol{pop}}}{\boldsymbol{N}_{\boldsymbol{all}}\boldsymbol{\times}\boldsymbol{Biovolume}_{\boldsymbol{all}}}$ (1)

where *F_pop_* represents the fraction of biovolume of a specific phytoplankton population (*pop*) relative to total phytoplankton biovolume and *N* represents the abundance via cell count of a specific population or all phytoplankton cells (*all*). Biovolume was calculated using area-integrated forward scatter (*FSC-A*) converted with a power regression [11] as follows:

$\boldsymbol{Biovolume (}\boldsymbol{\mu m}^{\boldsymbol{3}}\boldsymbol{)=}\mathbf{exp}\boldsymbol{(}\frac{\log\left( \boldsymbol{FSC-A} \right)\boldsymbol{-}\log\left( \boldsymbol{10248} \right)}{\boldsymbol{0.4653}}\boldsymbol{)}$ (2)

Cell diameter and surface area were calculated from biovolume assuming that cells had a spherical geometry. Biovolume was converted to C biomass using the following equation from Menden-Deuer and Lessar (2000) [2]:

$\boldsymbol{C}\left( \boldsymbol{pgC}\boldsymbol{cell}^{\boldsymbol{-1}} \right)\boldsymbol{=0.216 \times}\boldsymbol{biovolume}^{\boldsymbol{0.939}}$(3)

Statistical analyses. We used linear mixed effect models to examine the effects of size fractions, light, and pre- versus post-filtration on the tested response variables using R (R “stats” packages) [12] (Dataset S1). Models were fit with the ‘lme’ function of the ‘lme4’ package, using maximum likelihood. The variability between replicates (or bottle effect) was included as a random effect in all analyses. Using the ‘drop1’ function with a Chi-squared test (null hypothesis of independence), the best model fit was selected by sequentially eliminating variables among the fixed effects: size fraction / plankton group, pre-filtration, time, and light. To normalize model residuals, data were log_10_ transformed before analyses. Addition treatments (e.g. light, pre-filtration) were treated as two separate factors, each possessing two levels (‘True’ or ‘False’). When treatment effects were suggested by the model fit (with more than two factor levels), we performed pairwise comparisons using the ‘emmeans’ package with the Tukey method. All statements of significance (*P* value <0.05) refer to the linear mixed effect models results and pairwise comparisons.

**SI References**

1. Holte, J, Talley, L. A new algorithm for finding mixed layer depths with applications to argo data and subantarctic mode water formation. J Atmos Oceanic Technol 2009; 26: 1920-1939. doi: 10.1175/2009JTECHO543.1.

2. Menden-Deuer, S, Lessard, EJ. Carbon to volume relationships for dinoflagellates, diatoms, and other protist plankton. Limnol Oceanogr 2000; 45: 569-579. doi: 10.4319/lo.2000.45.3.0569.

3. Gundersen, K, Heldal, M, Norland, S, Purdie, DA, Knap, AH. Elemental C, N, and P cell content of individual bacteria collected at the Bermuda Atlantic Time‐series Study (BATS) site. Limnol Oceanogr 2002; 47: 1525-1530. doi: 10.4319/lo.2002.47.5.1525.

4. Fukuda, R, Ogawa, H, Nagata, T, Koike, I. Direct determination of carbon and nitrogen contents of natural bacterial assemblages in marine environments. Appl Environ Microbiol 1998; 64: 3352-3358. doi: 10.1128/AEM.64.9.3352-3358.1998.

5. Booth, BC. Size classes and major taxonomic groups of phytoplankton at two locations in the subarctic Pacific Ocean in May and August, 1984. Mar Biol 1988; 97: 275-286. doi: 10.1007/BF00391313.

6. Geider, RJ. Estimating the growth and loss rates of phytoplankton from time-series observations of 14C-bicarbonate uptake. Mar Ecol Prog Ser 1988; 43: 125-138.

7. Fourquez, M, Bressac, M, Deppeler, SL, Ellwood, M, Obernosterer, I, Trull, TW, et al. Microbial Competition in the Subpolar Southern Ocean: An Fe–C Co-limitation Experiment. Front Mar Sci 2020; 6: 776. doi: 10.3389/fmars.2019.00776.

8. Shaked, Y, Twining, BS, Tagliabue, A, Maldonado, MT. Probing the bioavailability of dissolved iron to marine eukaryotic phytoplankton using in situ single cell iron quotas. Global Biogeochem Cycles. 2021; 35: e2021GB006979. doi: 10.1029/2021GB006979.

9. Marie, D, Partensky, F, Vaulot, D, Brussaard, C. Enumeration of phytoplankton, bacteria, and viruses in marine samples. Curr Protoc Cytom 1999; 10. doi: 10.1002/0471142956.cy1111s10.

10. Bach, LT, Lohbeck, KT, Reusch, TBH, Riebesell, U. Rapid evolution of highly variable competitive abilities in a key phytoplankton species. Nat Ecol Evol 2018; 2: 611-613. doi: 10.1038/s41559-018-0474-x.

11. Selfe C. Developing transfer function to measuring phytoplankton cellular properties with flow cytometry. [Master’s thesis]. Hobart, Univ. of Tasmania (Australia): Institute for Marine and Antarctic Studies; 2022.

12. R Core Team. R: A language and environment for statistical computing. R Foundation for Statistical Computing; 2020. Available from: https://www.R-project.org/.
