# Supplementary material for: Microbial competition for iron determines its availability to the ferrous wheel
Source: ISME J. 2025 Jan 27;19(1):wraf015. doi: 10.1093/ismejo/wraf015 (PMC11833320; doi:10.1093/ismejo/wraf015)
Supplement: Strzepek_ISME_DatasetS1_wraf015 [file strzepek_isme_datasets1_wraf015.pdf]

Microbial competition for iron determines its availability to the Ferrous Wheel

Robert F. Strzepek, Pauline Latour, Michael J. Ellwood, Yeala Shaked, Philip W. Boyd

Dataset S1: Statistical Analyses

Contents

1 Background 1

2 Fe uptake in all fractions 2

2.1 ANOVA test 2

2.2 LMM test 3

3 Carbon uptake in all fractions 8

3.1 ANOVA test 8

3.2 LMM test 9

4 Fe:C ratios in all fractions 13

4.1 ANOVA test 13

4.2 LMM test 14

5 Fe uptake in picoeukaryotes vs bacteria 19

5.1 t-test 19

5.2 LMM test 20

6 Carbon uptake in picoeukaryotes vs bacteria 25

6.1 t-test 25

6.2 LMM test 26

7 Fe uptake with pre-filtration and light 30

7.1 LMM test 30

1 Background

This R markdown document shows the detailed results of the statistical analyses on our manuscript: 'Microbial competition for iron in the Southern Ocean'. To study the effect of size fraction, light conditions and pre-filtration on iron/carbon uptake and subsequent Fe:C ratios from Southern Ocean microbial communities, we performed linear mixed effect model and/or t-test. The R code and output results can be found below.

2 Fe uptake in all fractions

```
Fe_upt_ini <- read.csv("Fractions_Fe.C_PL.csv")
```

2.1 ANOVA test

```
#ANOVA to compare 3 or more means (because LMM might be overkill)
res.aov <- aov(Fe.uptake~Fraction, data = Fe_upt_ini)
summary(res.aov)
```

```
##             Df Sum Sq Mean Sq F value Pr(>F)
## Fraction     3  619.5   206.5   8.164 0.0081 **
## Residuals    8  202.4    25.3
## ---
## Signif. codes:  0 '***' 0.001 '**' 0.01 '*' 0.05 '.' 0.1 ' ' 1
```

```
#it shows there is significant difference in Fe uptake between some fractions

#Check homogeneity of data:
leveneTest(Fe.uptake~Fraction, data=Fe_upt_ini)
```

```
## Levene's Test for Homogeneity of Variance (center = median)
##       Df F value Pr(>F)
## group  3  0.8209  0.518
##        8
```

```
#p-value > 0.05 so we can assume homogeneity of variances in the different fractions

#We also want to check if the distribution is normal
#extract residuals
aov_residuals <- residuals(object=res.aov)
#Shapiro-Wilk test
shapiro.test(x=aov_residuals)
```

```
##
##  Shapiro-Wilk normality test
##
## data:  aov_residuals
## W = 0.9238, p-value = 0.319
```

```
#p-value > 0.05, normality is respected.

#Tukey test for multiple pairwise-comparisons
TukeyHSD(res.aov)
```

```
##   Tukey multiple comparisons of means
##     95% family-wise confidence level
##
```

```
## Fit: aov(formula = Fe.uptake ~ Fraction, data = Fe_upt_ini)
##
## $Fraction
##                     diff         lwr        upr     p adj
## 0.2-0.8->20     5.686667  -7.4639140 18.8372473 0.5411289
## 0.8-2->20      19.656667   6.5060860 32.8072473 0.0060153
## 2-20->20        6.866667  -6.2839140 20.0172473 0.3957639
## 0.8-2-0.2-0.8  13.970000   0.8194193 27.1205807 0.0377998
## 2-20-0.2-0.8    1.180000 -11.9705807 14.3305807 0.9910912
## 2-20-0.8-2    -12.790000 -25.9405807  0.3605807 0.0565835
```

```
#it shows significant difference between 0.8-2um and >20um + 0.8-2um and 0.2-0.8
```

2.2 LMM test

```
Fe_upt_ini$ln_Fe <- log(Fe_upt_ini$Fe.uptake)   #data log-transformed
```

```
#Plot using ggplot2
ggplot(Fe_upt_ini) +
  geom_point(aes(x = Fraction, y = ln_Fe)) +
  scale_y_log10()
```

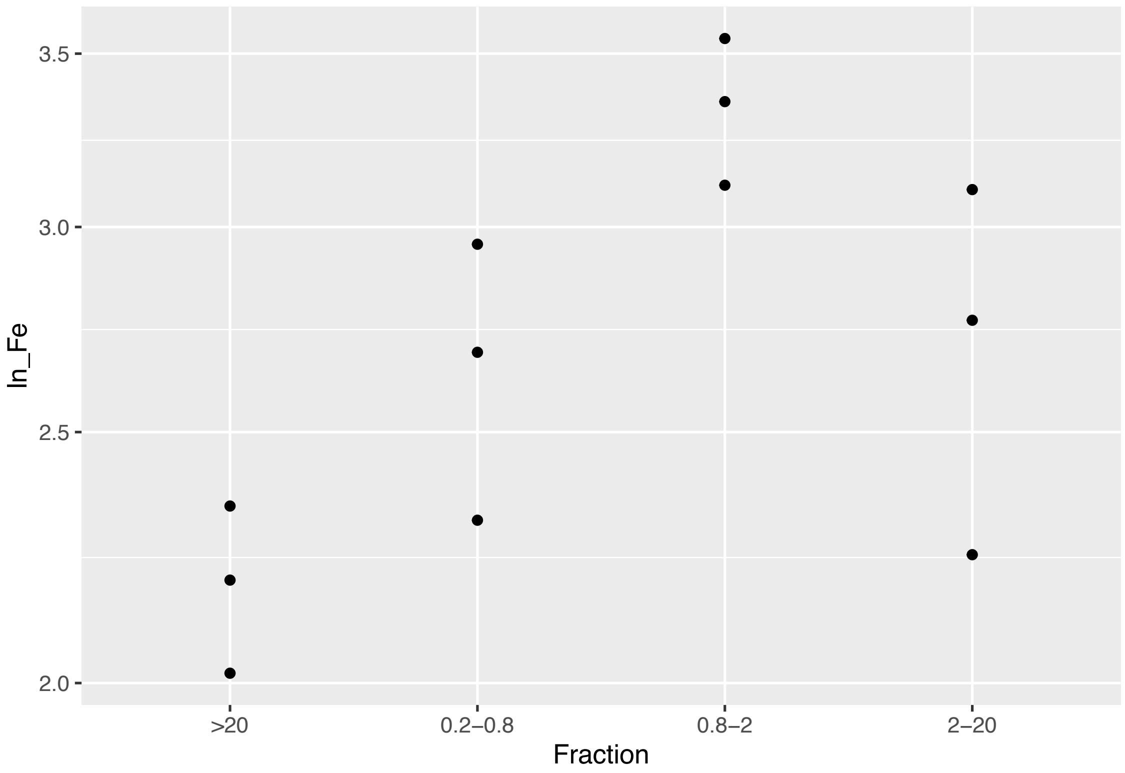

```r
#Model fit with fraction
fit_Feini <- lmer(ln_Fe ~ Fraction + (1|Bottle),
               data = Fe_upt_ini, na.action=na.fail, REML = FALSE)

summary(fit_Feini)
```

```
## Linear mixed model fit by maximum likelihood  ['lmerMod']
## Formula: ln_Fe ~ Fraction + (1 | Bottle)
##    Data: Fe_upt_ini
##
##      AIC      BIC   logLik deviance df.resid
##     12.5     15.4     -0.2      0.5        6
##
## Scaled residuals:
##     Min      1Q  Median      3Q     Max
## -1.8639 -0.7318  0.1002  0.6908  1.6215
##
## Random effects:
##  Groups   Name        Variance Std.Dev.
##  Bottle   (Intercept) 0.00000  0.0000
##  Residual             0.06084  0.2466
## Number of obs: 12, groups:  Bottle, 3
##
## Fixed effects:
##                 Estimate Std. Error t value
## (Intercept)       2.1834     0.1424  15.332
## Fraction0.2-0.8   0.4665     0.2014   2.317
## Fraction0.8-2     1.1548     0.2014   5.734
## Fraction2-20      0.5181     0.2014   2.573
##
## Correlation of Fixed Effects:
##             (Intr) F0.2-0 F0.8-2
## Frct0.2-0.8 -0.707
## Fractn0.8-2 -0.707  0.500
## Fractin2-20 -0.707  0.500  0.500
## optimizer (nloptwrap) convergence code: 0 (OK)
## boundary (singular) fit: see help('isSingular')
```

```r
#drop function perform valid likelihood ratio tests
drop1(fit_Feini, test = "Chisq")
```

```
## Single term deletions
##
## Model:
## ln_Fe ~ Fraction + (1 | Bottle)
##          npar    AIC    LRT  Pr(Chi)
## <none>        12.460
## Fraction    3 22.402 15.942 0.001165 **
## ---
## Signif. codes:  0 '***' 0.001 '**' 0.01 '*' 0.05 '.' 0.1 ' ' 1
```

```r
#it shows that 'fraction' significantly influences Fe uptake

#Report results
report(fit_Feini)
```

```
## Random effect variances not available. Returned R2 does not account for random effects.
## Random effect variances not available. Returned R2 does not account for random effects.
```

```
## We fitted a linear mixed model (estimated using ML and nloptwrap optimizer) to
## predict ln_Fe with Fraction (formula: ln_Fe ~ Fraction). The model included
## Bottle as random effect (formula: ~1 | Bottle). The model's explanatory power
## related to the fixed effects alone (marginal R2) is 0.75. The model's
## intercept, corresponding to Fraction = >20, is at 2.18 (95% CI [1.83, 2.53],
## t(6) = 15.33, p < .001). Within this model:
##
##   - The effect of Fraction [0.2-0.8] is statistically non-significant and
## positive (beta = 0.47, 95% CI [-0.03, 0.96], t(6) = 2.32, p = 0.060; Std. beta
## = 0.93, 95% CI [-0.05, 1.92])
##   - The effect of Fraction [0.8-2] is statistically significant and positive
## (beta = 1.15, 95% CI [0.66, 1.65], t(6) = 5.73, p = 0.001; Std. beta = 2.31,
## 95% CI [1.32, 3.29])
##   - The effect of Fraction [2-20] is statistically significant and positive (beta
## = 0.52, 95% CI [0.03, 1.01], t(6) = 2.57, p = 0.042; Std. beta = 1.04, 95% CI
## [0.05, 2.02])
##
## Standardized parameters were obtained by fitting the model on a standardized
## version of the dataset. 95% Confidence Intervals (CIs) and p-values were
## computed using a Wald t-distribution approximation.
```

```r
#Represent data predicted by the model vs real data
Fe_upt_ini$PredictedFe <- exp(predict(fit_Feini, re.form = NA))
```

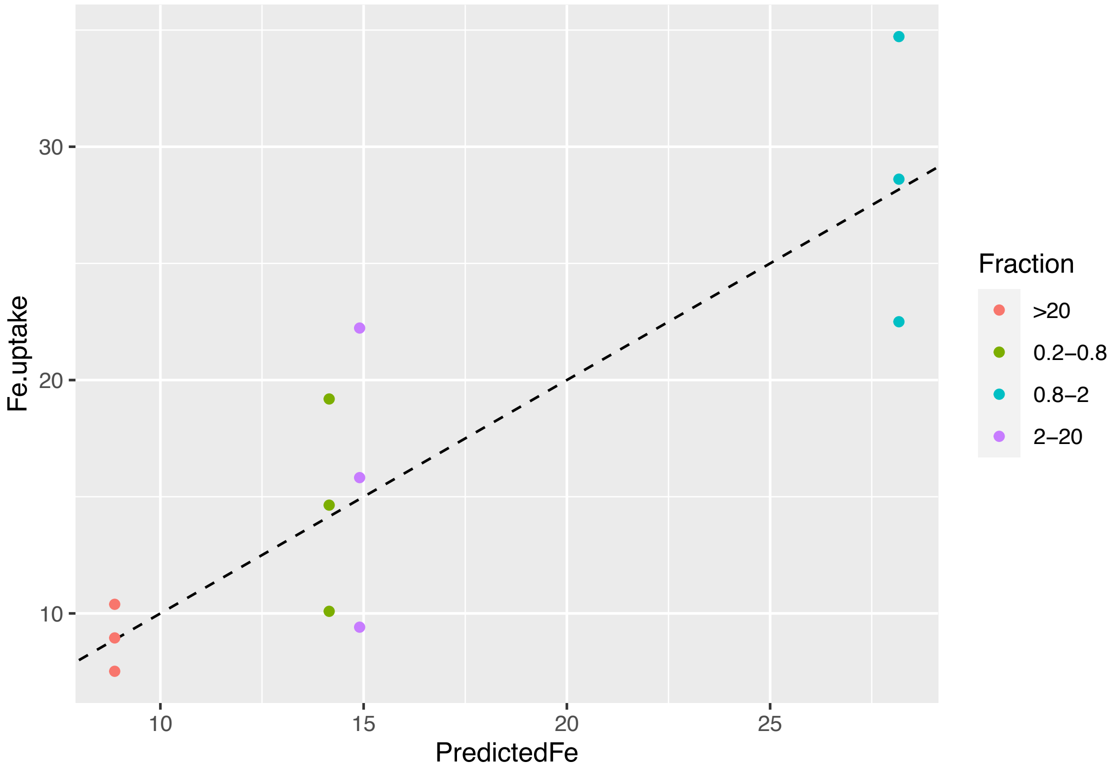

Fe.uptake
30
20
10
10
15
20
25
PredictedFe
Fraction
>20
0.2–0.8
0.8–2
2–20

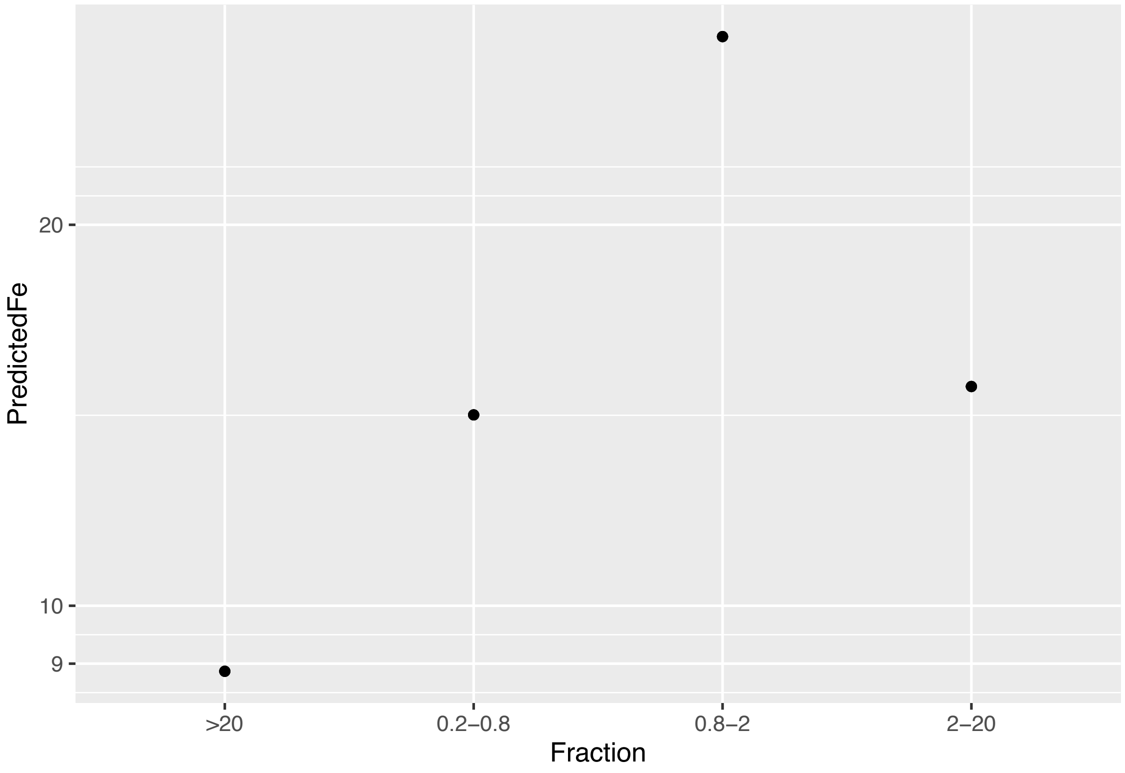


```r
#Pairwise comparison
emmeans (fit_Feini, pairwise ~ Fraction, adjust = "tukey")
```

```
## boundary (singular) fit: see help('isSingular')

## $emmeans
##  Fraction emmean    SE df lower.CL upper.CL
##  >20        2.18 0.174 18     1.82     2.55
##  0.2-0.8    2.65 0.174 18     2.28     3.02
##  0.8-2      3.34 0.174 18     2.97     3.70
##  2-20       2.70 0.174 18     2.34     3.07
##
## Degrees-of-freedom method: kenward-roger
## Confidence level used: 0.95
##
## $contrasts
##  contrast            estimate    SE   df t.ratio p.value
##  >20 - (0.2-0.8)      -0.4665 0.247 13.5  -1.892  0.2767
##  >20 - (0.8-2)        -1.1548 0.247 13.5  -4.682  0.0019
##  >20 - (2-20)         -0.5181 0.247 13.5  -2.101  0.2020
##  (0.2-0.8) - (0.8-2)  -0.6883 0.247 13.5  -2.791  0.0631
##  (0.2-0.8) - (2-20)   -0.0516 0.247 13.5  -0.209  0.9966
##  (0.8-2) - (2-20)      0.6367 0.247 13.5   2.581  0.0912
##
## Degrees-of-freedom method: kenward-roger
## P value adjustment: tukey method for comparing a family of 4 estimates
```

3 Carbon uptake in all fractions

3.1 ANOVA test

```
#ANOVA to compare 3 or more means (because LMM might be overkill)
res.aov <- aov(C.uptake~Fraction, data = Fe_upt_ini)
summary(res.aov)
```

```
##             Df Sum Sq Mean Sq F value   Pr(>F)
## Fraction     3  5.511  1.8372   76.34 3.15e-06 ***
## Residuals    8  0.193  0.0241
## ---
## Signif. codes:  0 '***' 0.001 '**' 0.01 '*' 0.05 '.' 0.1 ' ' 1
```

```
#it shows there is significant difference in C uptake between some fractions

#Check homogeneity of data:
leveneTest(C.uptake~Fraction, data=Fe_upt_ini)
```

```
## Levene's Test for Homogeneity of Variance (center = median)
##       Df F value Pr(>F)
## group  3  2.0041  0.192
##        8
```

```
#p-value > 0.05 so we can assume homogeneity of variances in the different fractions

#We also want to check if the distribution is normal
#extract residuals
aov_residuals <- residuals(object=res.aov)
#Shapiro-Wilk test
shapiro.test(x=aov_residuals)
```

```
##
##  Shapiro-Wilk normality test
##
## data:  aov_residuals
## W = 0.9321, p-value = 0.4029
```

```
#p-value > 0.05, normality is respected.

#Tukey test for multiple pairwise-comparisons
TukeyHSD(res.aov)
```

```
##   Tukey multiple comparisons of means
##     95% family-wise confidence level
##
## Fit: aov(formula = C.uptake ~ Fraction, data = Fe_upt_ini)
##
## $Fraction
##                      diff          lwr        upr     p adj
```

```
## 0.2-0.8->20   -1.7733333 -2.178964587 -1.3677021 0.0000031
## 0.8-2->20     -1.5166667 -1.922297921 -1.1110354 0.0000102
## 2-20->20      -1.1133333 -1.518964587 -0.7077021 0.0001021
## 0.8-2-0.2-0.8  0.2566667 -0.148964587  0.6622979 0.2551927
## 2-20-0.2-0.8   0.6600000  0.254368746  1.0656313 0.0035878
## 2-20-0.8-2     0.4033333 -0.002297921  0.8089646 0.0512930
```

```
#it shows significant difference in C uptake between all fractions except 0.8-2um/0.2-0.8um
#and 2-20um/0.8-2um
```

3.2 LMM test

```
Fe_upt_ini$ln_C <- log(Fe_upt_ini$C.uptake)   #data log-transformed
#but log-transforming the data result in negative values
#so I kept the normal values and did not use the log
```

```
#Plot using ggplot2
ggplot(Fe_upt_ini) +
  geom_point(aes(x = Fraction, y = C.uptake)) +
  scale_y_log10()
```

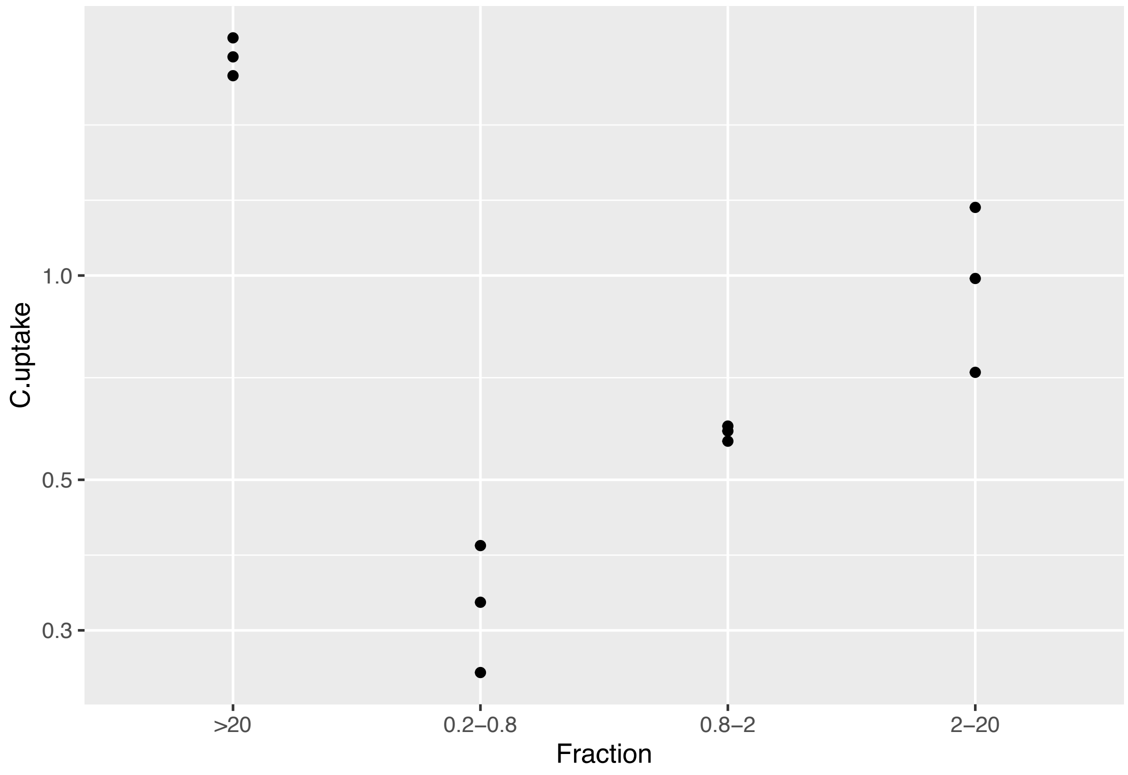

```r
#Model fit with fraction
fit_Cini <- lmer(C.uptake ~ Fraction + (1|Bottle),
               data = Fe_upt_ini, na.action=na.fail, REML = FALSE)

summary(fit_Cini)
```

```
## Linear mixed model fit by maximum likelihood  ['lmerMod']
## Formula: C.uptake ~ Fraction + (1 | Bottle)
##    Data: Fe_upt_ini
##
##      AIC      BIC   logLik deviance df.resid
##     -4.0     -1.1      8.0    -16.0        6
##
## Scaled residuals:
##      Min       1Q   Median       3Q      Max
## -1.92477 -0.42432 -0.02441  0.45764  2.00741
##
## Random effects:
##  Groups   Name        Variance Std.Dev.
##  Bottle   (Intercept) 0.002941 0.05423
##  Residual             0.013104 0.11447
## Number of obs: 12, groups:  Bottle, 3
##
## Fixed effects:
##                  Estimate Std. Error t value
## (Intercept)       2.10333    0.07313   28.76
## Fraction0.2-0.8 -1.77333    0.09347  -18.97
## Fraction0.8-2   -1.51667    0.09347  -16.23
## Fraction2-20    -1.11333    0.09347  -11.91
##
## Correlation of Fixed Effects:
##             (Intr) F0.2-0 F0.8-2
## Frct0.2-0.8 -0.639
## Fractn0.8-2 -0.639  0.500
## Fractin2-20 -0.639  0.500  0.500
```

```r
#drop function perform valid likelihood ratio tests
drop1(fit_Cini, test = "Chisq")
```

```
## Single term deletions
##
## Model:
## C.uptake ~ Fraction + (1 | Bottle)
##          npar     AIC    LRT   Pr(Chi)
## <none>        -4.0419
## Fraction    3 31.1297 41.172 6.013e-09 ***
## ---
## Signif. codes:  0 '***' 0.001 '**' 0.01 '*' 0.05 '.' 0.1 ' ' 1
```

```r
#it shows that 'fraction' significantly influences C uptake

#Report results
report(fit_Cini)
```

```
## We fitted a linear mixed model (estimated using ML and nloptwrap optimizer) to
## predict C.uptake with Fraction (formula: C.uptake ~ Fraction). The model
## included Bottle as random effect (formula: ~1 | Bottle). The model's total
## explanatory power is substantial (conditional R2 = 0.97) and the part related
## to the fixed effects alone (marginal R2) is of 0.97. The model's intercept,
## corresponding to Fraction = >20, is at 2.10 (95% CI [1.92, 2.28], t(6) = 28.76,
## p < .001). Within this model:
##
##   - The effect of Fraction [0.2-0.8] is statistically significant and negative
## (beta = -1.77, 95% CI [-2.00, -1.54], t(6) = -18.97, p < .001; Std. beta =
## -2.46, 95% CI [-2.78, -2.15])
##   - The effect of Fraction [0.8-2] is statistically significant and negative
## (beta = -1.52, 95% CI [-1.75, -1.29], t(6) = -16.23, p < .001; Std. beta =
## -2.11, 95% CI [-2.42, -1.79])
##   - The effect of Fraction [2-20] is statistically significant and negative (beta
## = -1.11, 95% CI [-1.34, -0.88], t(6) = -11.91, p < .001; Std. beta = -1.55, 95%
## CI [-1.86, -1.23])
##
## Standardized parameters were obtained by fitting the model on a standardized
## version of the dataset. 95% Confidence Intervals (CIs) and p-values were
## computed using a Wald t-distribution approximation.
```

```r
#Represent data predicted by the model vs real data
Fe_upt_ini$PredictedC <- exp(predict(fit_Cini, re.form = NA))
```

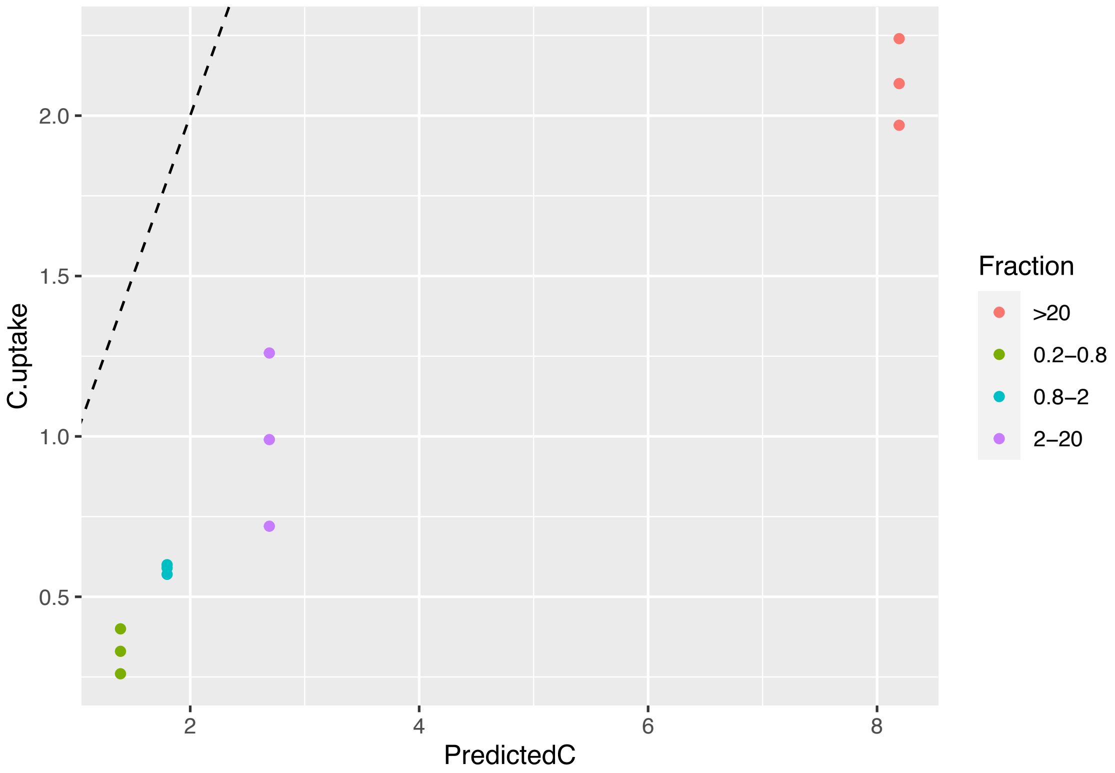

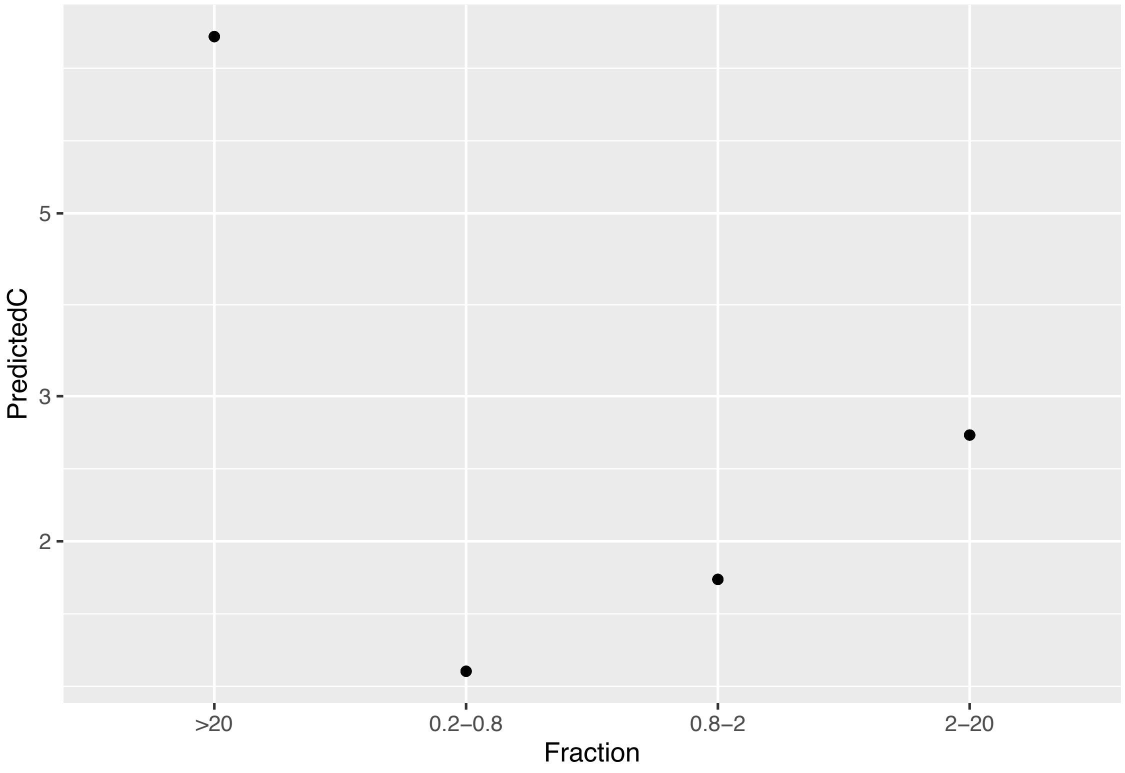


```r
#Pairwise comparison
emmeans (fit_Cini, pairwise ~ Fraction, adjust = "tukey")
```

```
## $emmeans
##  Fraction emmean     SE   df lower.CL upper.CL
##  >20       2.103 0.0896 16.4    1.914    2.293
##  0.2-0.8   0.330 0.0896 16.4    0.140    0.520
##  0.8-2     0.587 0.0896 16.4    0.397    0.776
##  2-20      0.990 0.0896 16.4    0.800    1.180
##
## Degrees-of-freedom method: kenward-roger
## Confidence level used: 0.95
##
## $contrasts
##  contrast             estimate    SE   df t.ratio p.value
##  >20 - (0.2-0.8)         1.773 0.114 13.5  15.491  <.0001
##  >20 - (0.8-2)           1.517 0.114 13.5  13.249  <.0001
##  >20 - (2-20)            1.113 0.114 13.5   9.726  <.0001
##  (0.2-0.8) - (0.8-2)   -0.257 0.114 13.5  -2.242  0.1614
##  (0.2-0.8) - (2-20)    -0.660 0.114 13.5  -5.766  0.0003
##  (0.8-2) - (2-20)      -0.403 0.114 13.5  -3.523  0.0165
##
## Degrees-of-freedom method: kenward-roger
## P value adjustment: tukey method for comparing a family of 4 estimates
```

```
#it shows that all fractions are significantly different except 0.2-0.8um/0.8-0.2um
```

4 Fe:C ratios in all fractions

4.1 ANOVA test

```
#ANOVA to compare 3 or more means (because LMM might be overkill)
res.aov <- aov(Fe.C.uptake~Fraction, data = Fe_upt_ini)
summary(res.aov)
```

```
##             Df Sum Sq Mean Sq F value   Pr(>F)
## Fraction     3   4180    1393    34.8 6.13e-05 ***
## Residuals    8    320      40
## ---
## Signif. codes:  0 '***' 0.001 '**' 0.01 '*' 0.05 '.' 0.1 ' ' 1
```

```
#it shows there is significant difference in Fe:C ratios between fractions

#Check homogeneity of data:
leveneTest(Fe.C.uptake~Fraction, data=Fe_upt_ini)
```

```
## Levene's Test for Homogeneity of Variance (center = median)
##       Df F value Pr(>F)
## group  3  2.2994 0.1541
##        8
```

```
#p-value > 0.05 so we can assume homogeneity of variances in the different fractions

#We also want to check if the distribution is normal
#extract residuals
aov_residuals <- residuals(object=res.aov)
#Shapiro-Wilk test
shapiro.test(x=aov_residuals)
```

```
##
##  Shapiro-Wilk normality test
##
## data:  aov_residuals
## W = 0.90354, p-value = 0.1762
```

```
#p-value > 0.05, normality is respected.

#Tukey test for multiple pairwise-comparisons
TukeyHSD(res.aov)
```

```
##   Tukey multiple comparisons of means
##     95% family-wise confidence level
##
```

```
## Fit: aov(formula = Fe.C.uptake ~ Fraction, data = Fe_upt_ini)
##
## $Fraction
##                      diff        lwr       upr     p adj
## 0.2-0.8->20    39.106667  22.562352  55.65098 0.0002978
## 0.8-2->20      44.683333  28.139019  61.22765 0.0001148
## 2-20->20       11.293333  -5.250981  27.83765 0.2066398
## 0.8-2-0.2-0.8   5.576667 -10.967648  22.12098 0.7107966
## 2-20-0.2-0.8  -27.813333 -44.357648 -11.26902 0.0029241
## 2-20-0.8-2    -33.390000 -49.934315 -16.84569 0.0008863
```

```
#it shows significant difference in Fe:C ratios between all fractions except 2-20um/>20um
#and 0.8-2um/0.2-0.8um
```

4.2 LMM test

```
Fe_upt_ini$ln_Fe.C <- log(Fe_upt_ini$Fe.C.uptake)   #data log-transformed
```

```
#Plot using ggplot2
ggplot(Fe_upt_ini) +
  geom_point(aes(x = Fraction, y = ln_Fe.C)) +
  scale_y_log10()
```

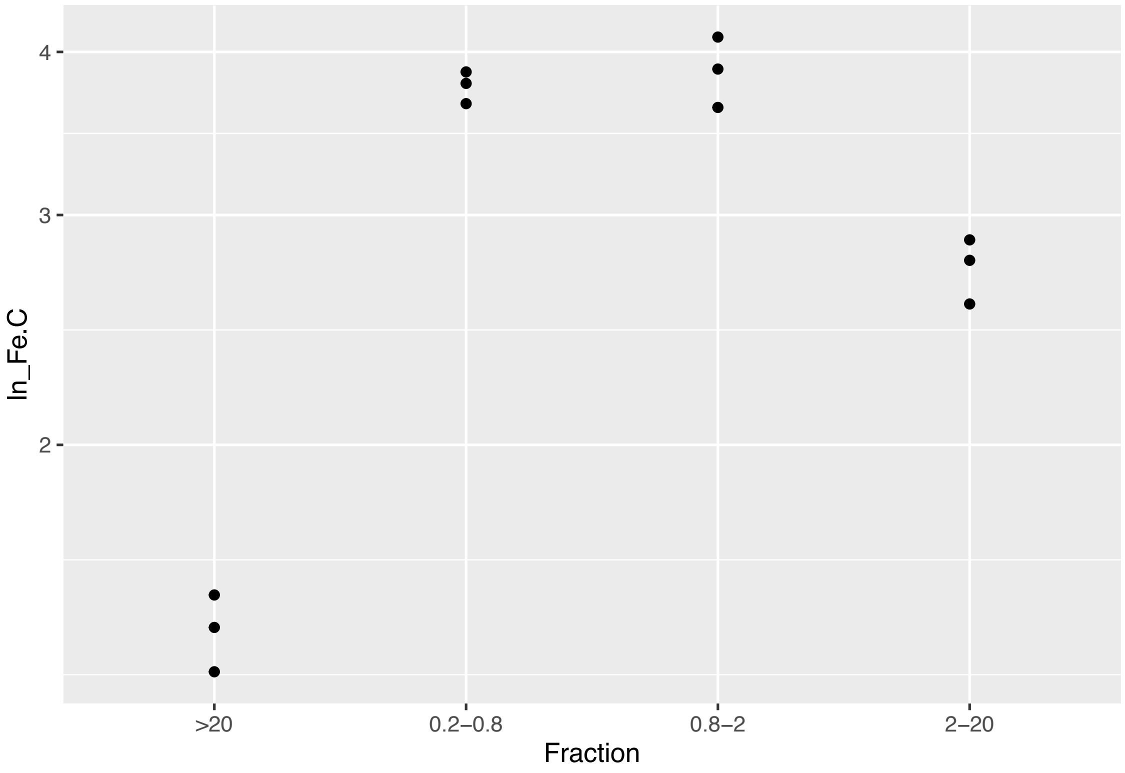

```r
#Model fit with fraction
fit_Fe.Cini <- lmer(ln_Fe.C ~ Fraction + (1|Bottle),
               data = Fe_upt_ini, na.action=na.fail, REML = FALSE)

summary(fit_Fe.Cini)
```

```
## Linear mixed model fit by maximum likelihood  ['lmerMod']
## Formula: ln_Fe.C ~ Fraction + (1 | Bottle)
##    Data: Fe_upt_ini
##
##      AIC      BIC   logLik deviance df.resid
##     -2.7      0.2      7.4    -14.7        6
##
## Scaled residuals:
##     Min      1Q  Median      3Q     Max
## -1.8682 -0.7980  0.1075  0.7177  1.7942
##
## Random effects:
##  Groups   Name        Variance Std.Dev.
##  Bottle   (Intercept) 0.00000  0.0000
##  Residual             0.01713  0.1309
## Number of obs: 12, groups:  Bottle, 3
##
## Fixed effects:
##                  Estimate Std. Error t value
## (Intercept)       1.44141    0.07557   19.07
## Fraction0.2-0.8  2.32409    0.10687   21.75
## Fraction0.8-2    2.42985    0.10687   22.74
## Fraction2-20     1.29357    0.10687   12.10
##
## Correlation of Fixed Effects:
##             (Intr) F0.2-0 F0.8-2
## Frct0.2-0.8 -0.707
## Fractn0.8-2 -0.707  0.500
## Fractin2-20 -0.707  0.500  0.500
## optimizer (nloptwrap) convergence code: 0 (OK)
## boundary (singular) fit: see help('isSingular')
```

```r
#drop function perform valid likelihood ratio tests
drop1(fit_Fe.Cini, test = "Chisq")
```

```
## Single term deletions
##
## Model:
## ln_Fe.C ~ Fraction + (1 | Bottle)
##          npar    AIC    LRT   Pr(Chi)
## <none>        -2.747
## Fraction    3 39.764 48.511 1.658e-10 ***
## ---
## Signif. codes:  0 '***' 0.001 '**' 0.01 '*' 0.05 '.' 0.1 ' ' 1
```

```r
#it shows that 'fraction' significantly influences C uptake

#Report results
report(fit_Fe.Cini)
```

```
## Random effect variances not available. Returned R2 does not account for random effects.
## Random effect variances not available. Returned R2 does not account for random effects.
```

```
## We fitted a linear mixed model (estimated using ML and nloptwrap optimizer) to
## predict ln_Fe.C with Fraction (formula: ln_Fe.C ~ Fraction). The model included
## Bottle as random effect (formula: ~1 | Bottle). The model's explanatory power
## related to the fixed effects alone (marginal R2) is 0.98. The model's
## intercept, corresponding to Fraction = >20, is at 1.44 (95% CI [1.26, 1.63],
## t(6) = 19.07, p < .001). Within this model:
##
##   - The effect of Fraction [0.2-0.8] is statistically significant and positive
## (beta = 2.32, 95% CI [2.06, 2.59], t(6) = 21.75, p < .001; Std. beta = 2.25,
## 95% CI [2.00, 2.51])
##   - The effect of Fraction [0.8-2] is statistically significant and positive
## (beta = 2.43, 95% CI [2.17, 2.69], t(6) = 22.74, p < .001; Std. beta = 2.35,
## 95% CI [2.10, 2.61])
##   - The effect of Fraction [2-20] is statistically significant and positive (beta
## = 1.29, 95% CI [1.03, 1.56], t(6) = 12.10, p < .001; Std. beta = 1.25, 95% CI
## [1.00, 1.51])
##
## Standardized parameters were obtained by fitting the model on a standardized
## version of the dataset. 95% Confidence Intervals (CIs) and p-values were
## computed using a Wald t-distribution approximation.
```

```r
#Represent data predicted by the model vs real data
Fe_upt_ini$PredictedFe.C <- exp(predict(fit_Fe.Cini, re.form = NA))
```

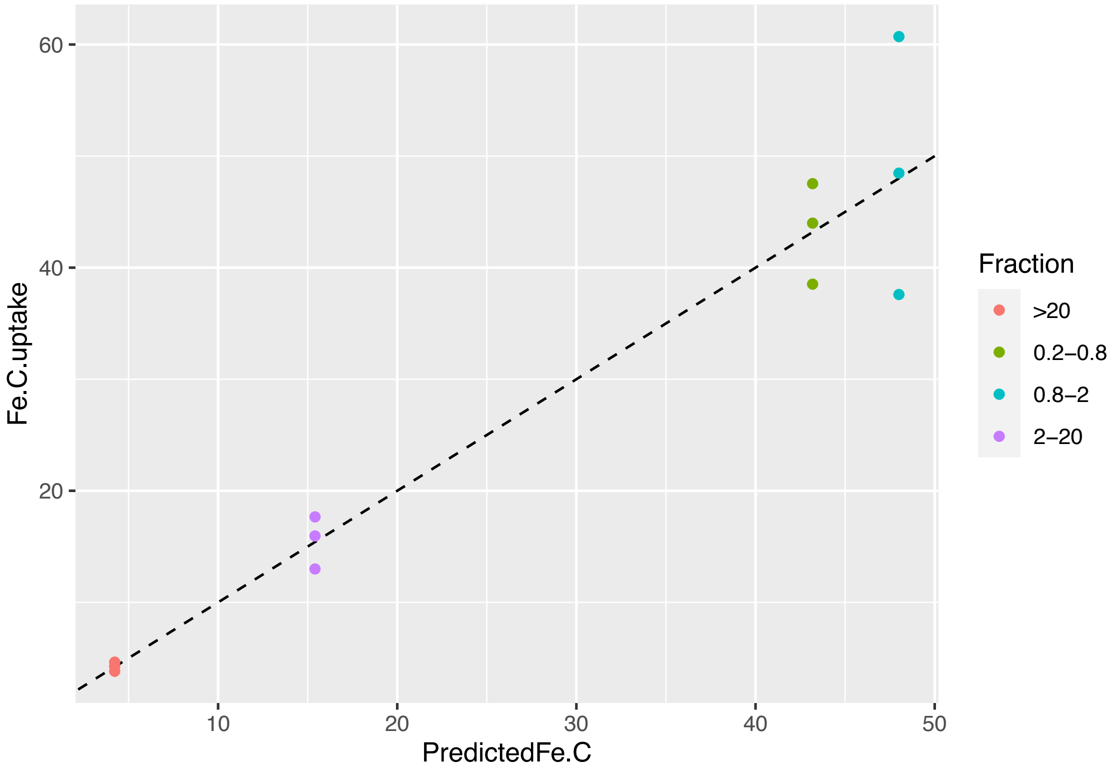
60
40
20
Fe.C.uptake
10
20
30
40
50
PredictedFe.C
Fraction
>20
0.2−0.8
0.8−2
2−20

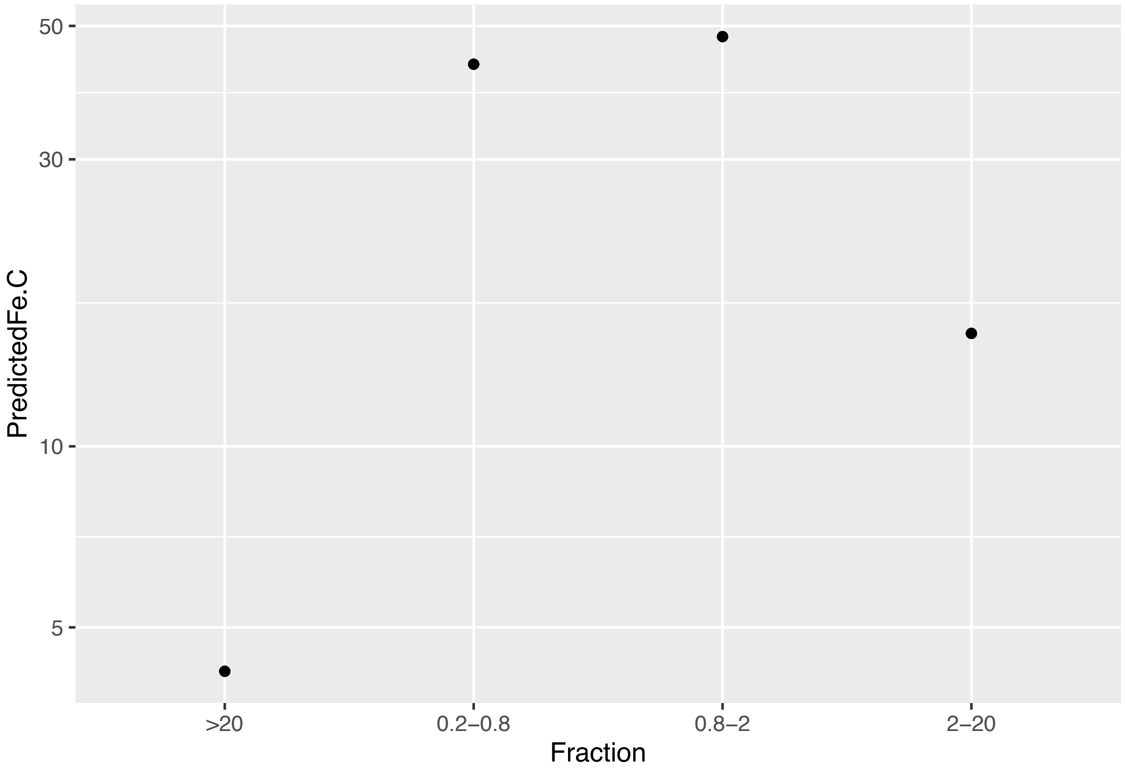


```r
#Pairwise comparison
emmeans (fit_Fe.Cini, pairwise ~ Fraction, adjust = "tukey")
```

```
## boundary (singular) fit: see help('isSingular')

## $emmeans
##  Fraction emmean     SE df lower.CL upper.CL
##  >20        1.44 0.0926 18     1.25     1.64
##  0.2-0.8    3.77 0.0926 18     3.57     3.96
##  0.8-2      3.87 0.0926 18     3.68     4.07
##  2-20       2.73 0.0926 18     2.54     2.93
##
## Degrees-of-freedom method: kenward-roger
## Confidence level used: 0.95
##
## $contrasts
##  contrast            estimate    SE   df t.ratio p.value
##  >20 - (0.2-0.8)       -2.324 0.131 13.5 -17.756  <.0001
##  >20 - (0.8-2)         -2.430 0.131 13.5 -18.564  <.0001
##  >20 - (2-20)          -1.294 0.131 13.5  -9.883  <.0001
##  (0.2-0.8) - (0.8-2)   -0.106 0.131 13.5  -0.808  0.8496
##  (0.2-0.8) - (2-20)     1.031 0.131 13.5   7.873  <.0001
##  (0.8-2) - (2-20)       1.136 0.131 13.5   8.681  <.0001
##
## Degrees-of-freedom method: kenward-roger
## P value adjustment: tukey method for comparing a family of 4 estimates
```

```r
#it shows that all fractions are significantly different except 0.2-0.8um/0.8-0.2um
```

5 Fe uptake in picoeukaryotes vs bacteria

```r
Fe_upt_pico <- read.csv("Fractions_Fe.C_secondfile_PL.csv")
```

5.1 t-test

```r
#simple t-test to compare 2 means (because LMM might be overkill)
#we first check variance of data
#F-test to check equal variance between groups
pico.ftest <- var.test(Fe.uptake ~ Fraction, data = Fe_upt_pico)
pico.ftest
```

```
##
##  F test to compare two variances
##
## data:  Fe.uptake by Fraction
## F = 0.017039, num df = 2, denom df = 2, p-value = 0.03351
## alternative hypothesis: true ratio of variances is not equal to 1
## 95 percent confidence interval:
##  0.0004368884 0.6645072676
## sample estimates:
## ratio of variances
##         0.01703865
```

```r
#it shows that p-value=0.03351 so there is significant difference between variance

#Welch t-test is used to compare means when equal variance is not assumed
pico.test <- t.test(Fe.uptake ~ Fraction, data=Fe_upt_pico, var.equal = FALSE)
pico.test
```

```
##
##  Welch Two Sample t-test
##
## data:  Fe.uptake by Fraction
## t = -7.3168, df = 2.0681, p-value = 0.01657
## alternative hypothesis: true difference in means between group 0.2-0.8 and group 0.8-2 is not equal
## 95 percent confidence interval:
##  -364.2627  -99.8046
## sample estimates:
## mean in group 0.2-0.8   mean in group 0.8-2
##              22.88067             254.91433
```

```r
#it shows Fe uptake is significantly different between pico and bacteria

#Check if data is normally distributed
```

```
library(ggpubr)
ggqqplot(Fe_upt_pico$Fe.uptake)
```

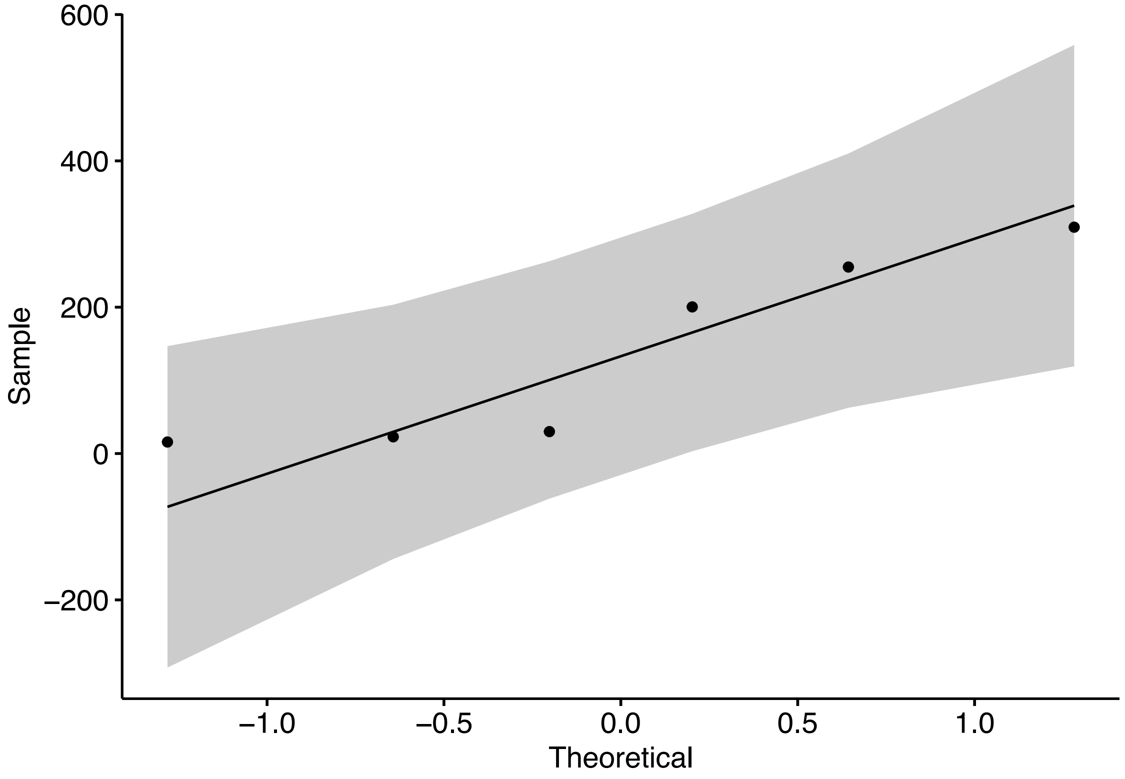


```
#or shapiro test
shapiro.test(Fe_upt_pico$Fe.uptake)
```

```
##
##  Shapiro-Wilk normality test
##
## data:  Fe_upt_pico$Fe.uptake
## W = 0.83347, p-value = 0.115
```

```
#it shows that yes data is normally distributed
```

5.2 LMM test

```
#But just in case we also do an LMM (account for bottle effect)
Fe_upt_pico$ln_Fe <- log(Fe_upt_pico$Fe.uptake)  #data log-transformed
```

```
#Plot using ggplot2
ggplot(Fe_upt_pico) +
  geom_point(aes(x = Fraction, y = ln_Fe)) +
  scale_y_log10()
```

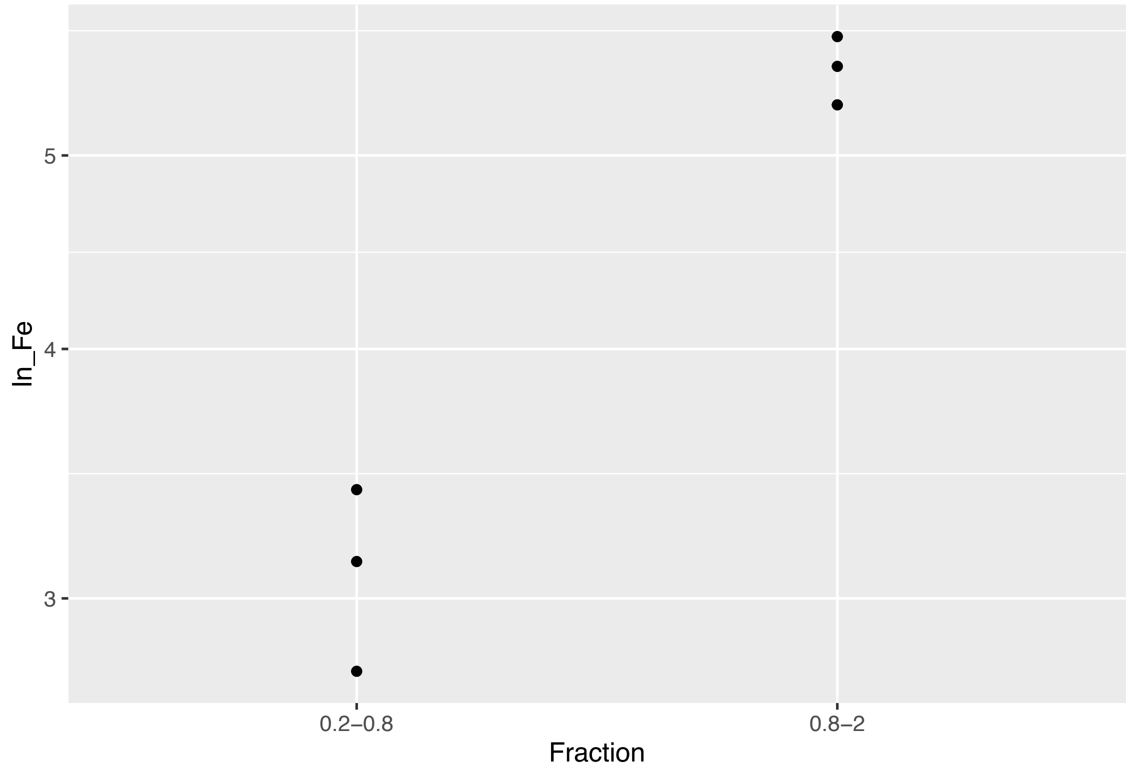


```r
#Model fit with fraction
fit_Fepico <- lmer(ln_Fe ~ Fraction + (1|Bottle),
               data = Fe_upt_pico, na.action=na.fail, REML = FALSE)

summary(fit_Fepico)
```

```
## Linear mixed model fit by maximum likelihood  ['lmerMod']
## Formula: ln_Fe ~ Fraction + (1 | Bottle)
##    Data: Fe_upt_pico
##
##      AIC      BIC   logLik deviance df.resid
##      7.1      6.3      0.4     -0.9        2
##
## Scaled residuals:
##     Min      1Q  Median      3Q     Max
## -1.5058 -0.7332  0.1100  0.7362  1.3551
##
## Random effects:
##  Groups   Name        Variance Std.Dev.
##  Bottle   (Intercept) 0.00000  0.0000
##  Residual             0.05047  0.2246
## Number of obs: 6, groups:  Bottle, 3
##
## Fixed effects:
##              Estimate Std. Error t value
## (Intercept)    3.0964     0.1297   23.87
```

```
## Fraction0.8-2   2.4289     0.1834   13.24
##
## Correlation of Fixed Effects:
##             (Intr)
## Fractn0.8-2 -0.707
## optimizer (nloptwrap) convergence code: 0 (OK)
## boundary (singular) fit: see help('isSingular')
```

```
#drop function perform valid likelihood ratio tests
drop1(fit_Fepico, test = "Chisq")
```

```
## Single term deletions
##
## Model:
## ln_Fe ~ Fraction + (1 | Bottle)
##          npar     AIC    LRT   Pr(Chi)
## <none>          7.1085
## Fraction    1 25.5606 20.452 6.114e-06 ***
## ---
## Signif. codes:  0 '***' 0.001 '**' 0.01 '*' 0.05 '.' 0.1 ' ' 1
```

```
#it shows that 'fraction' significantly influences Fe uptake

#Report results
report(fit_Fepico)
```

```
## Random effect variances not available. Returned R2 does not account for random effects.
## Random effect variances not available. Returned R2 does not account for random effects.
```

```
## We fitted a linear mixed model (estimated using ML and nloptwrap optimizer) to
## predict ln_Fe with Fraction (formula: ln_Fe ~ Fraction). The model included
## Bottle as random effect (formula: ~1 | Bottle). The model's explanatory power
## related to the fixed effects alone (marginal R2) is 0.97. The model's
## intercept, corresponding to Fraction = 0.2-0.8, is at 3.10 (95% CI [2.54,
## 3.65], t(2) = 23.87, p = 0.002). Within this model:
##
##   - The effect of Fraction [0.8-2] is statistically significant and positive
## (beta = 2.43, 95% CI [1.64, 3.22], t(2) = 13.24, p = 0.006; Std. beta = 1.80,
## 95% CI [1.21, 2.38])
##
## Standardized parameters were obtained by fitting the model on a standardized
## version of the dataset. 95% Confidence Intervals (CIs) and p-values were
## computed using a Wald t-distribution approximation.
```

```
#Represent data predicted by the model vs real data
Fe_upt_pico$Predicted <- exp(predict(fit_Fepico, re.form = NA))
```

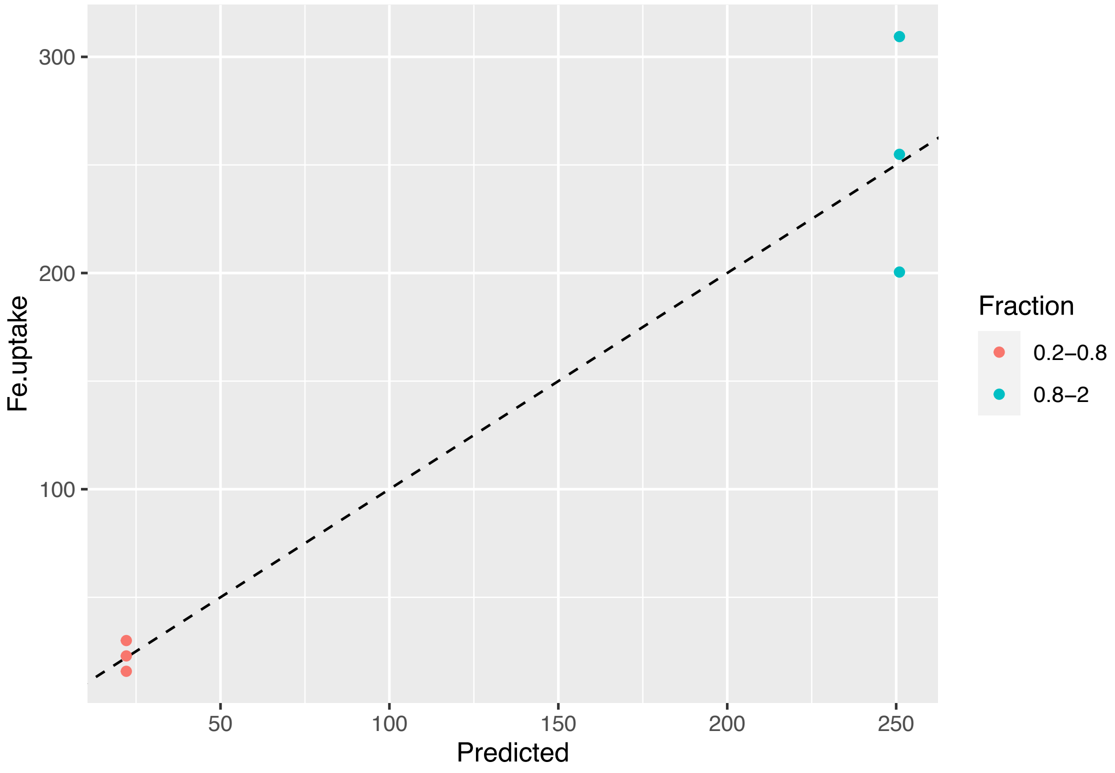
300
200
100
Fe.uptake
50
100
150
200
250
Predicted
Fraction
0.2–0.8
0.8–2

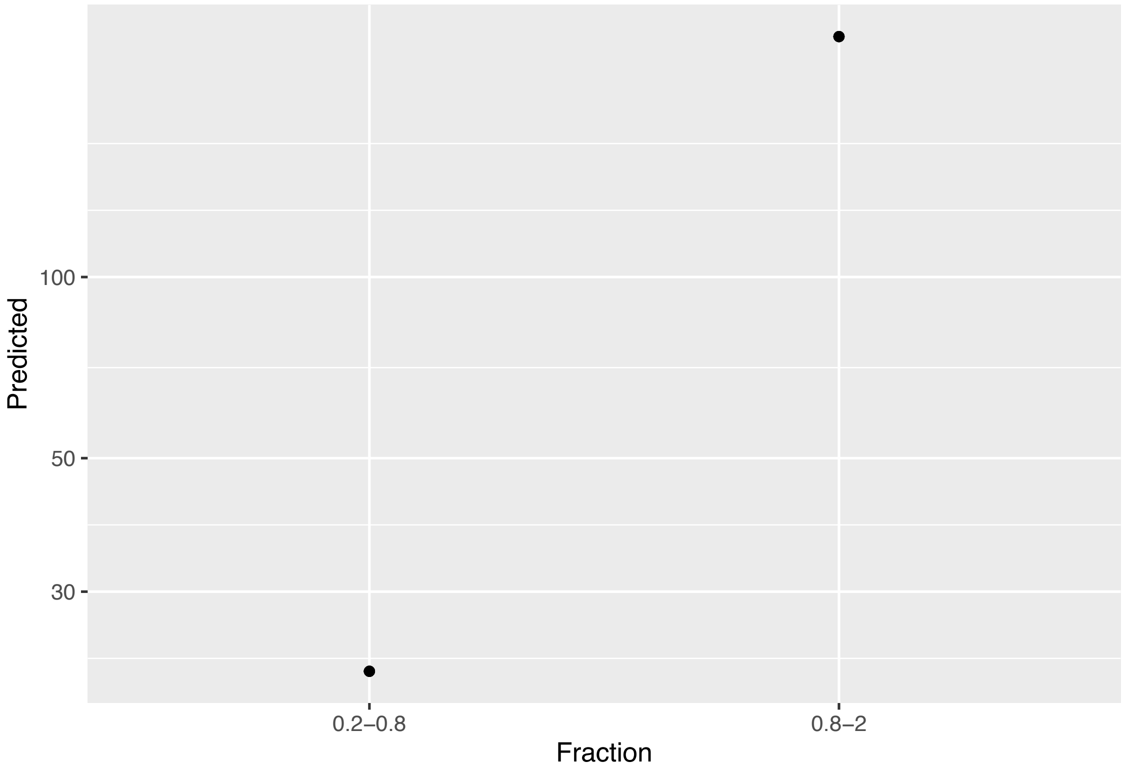


```r
#Pairwise comparison
emmeans (fit_Fepico, pairwise ~ Fraction, adjust = "tukey")
```

```
## boundary (singular) fit: see help('isSingular')
```

```
## $emmeans
##  Fraction emmean    SE df lower.CL upper.CL
##  0.2-0.8    3.10 0.159  9     2.74     3.46
##  0.8-2      5.53 0.159  9     5.17     5.88
##
## Degrees-of-freedom method: kenward-roger
## Confidence level used: 0.95
##
## $contrasts
##  contrast            estimate    SE  df t.ratio p.value
##  (0.2-0.8) - (0.8-2)    -2.43 0.225 4.5 -10.812  0.0002
##
## Degrees-of-freedom method: kenward-roger
```

6 Carbon uptake in picoeukaryotes vs bacteria

6.1 t-test

```
#simple t-test to compare 2 means (because LMM might be overkill)
#we first check variance of data
#F-test to check equal variance between groups
pico.ftest <- var.test(C.uptake ~ Fraction, data = Fe_upt_pico)
pico.ftest
```

```
##
##  F test to compare two variances
##
## data:  C.uptake by Fraction
## F = 0.83803, num df = 2, denom df = 2, p-value = 0.9119
## alternative hypothesis: true ratio of variances is not equal to 1
## 95 percent confidence interval:
##   0.02148795 32.68317954
## sample estimates:
## ratio of variances
##          0.8380302
```

```
#it shows that p-value=0.9 so there is no significant difference between variance

#Welch t-test is also used to compare means when normality is violated
pico.test <- t.test(C.uptake ~ Fraction, data=Fe_upt_pico, var.equal = FALSE)
pico.test
```

```
##
##  Welch Two Sample t-test
##
## data:  C.uptake by Fraction
## t = -49.619, df = 3.9692, p-value = 1.077e-06
## alternative hypothesis: true difference in means between group 0.2-0.8 and group 0.8-2 is not equal
## 95 percent confidence interval:
##  -4.973653 -4.445014
## sample estimates:
## mean in group 0.2-0.8   mean in group 0.8-2
##              0.520000              5.229333
```

```
#it shows C uptake is significantly different between pico and bacteria

#Check if data is normally distributed
library(ggpubr)
ggqqplot(Fe_upt_pico$C.uptake)
```

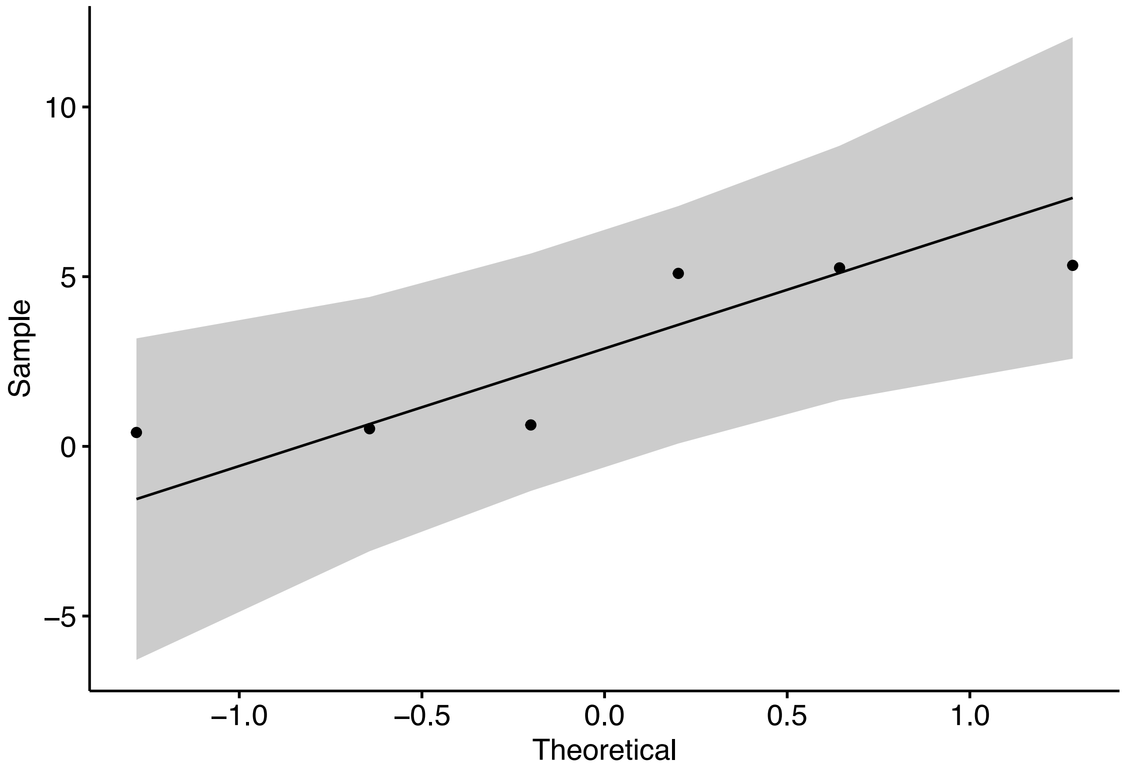


```
#or shapiro test
shapiro.test(Fe_upt_pico$C.uptake)
```

```
##
##  Shapiro-Wilk normality test
##
## data:  Fe_upt_pico$C.uptake
## W = 0.71773, p-value = 0.009454
```

```
#it shows that data distribution is NOT normal
```

6.2 LMM test

```
#But just in case we also do an LMM (account for bottle effect)
Fe_upt_pico$ln_C <- log(Fe_upt_pico$C.uptake)  #data log-transformed
#but again, same issue with negative values so model predict data wrongly!
```

```
#Plot using ggplot2
ggplot(Fe_upt_pico) +
  geom_point(aes(x = Fraction, y = C.uptake)) +
  scale_y_log10()
```

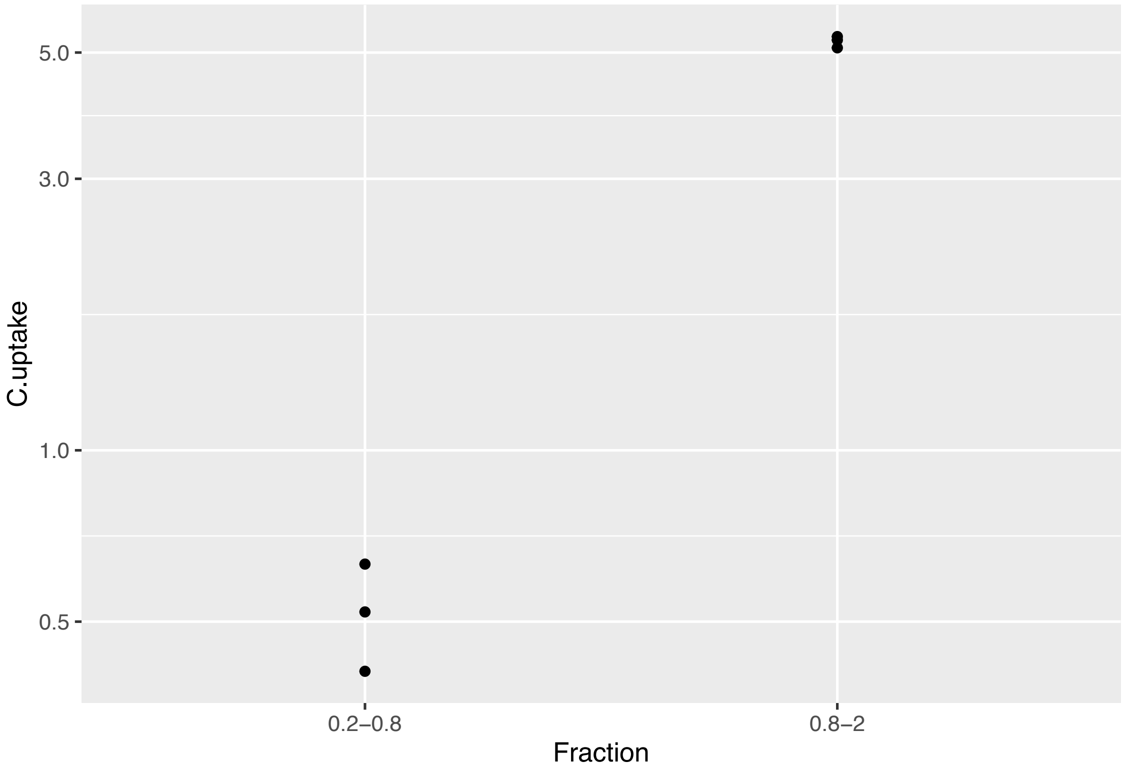


```r
#Model fit with fraction
fit_Cpico <- lmer(C.uptake ~ Fraction + (1|Bottle),
               data = Fe_upt_pico, na.action=na.fail, REML = FALSE)

summary(fit_Cpico)
```

```
## Linear mixed model fit by maximum likelihood  ['lmerMod']
## Formula: C.uptake ~ Fraction + (1 | Bottle)
##    Data: Fe_upt_pico
##
##      AIC      BIC   logLik deviance df.resid
##     -3.5     -4.4      5.8    -11.5        2
##
## Scaled residuals:
##     Min      1Q  Median      3Q     Max
## -1.2914 -0.7737  0.5033  0.6615  0.7699
##
## Random effects:
##  Groups   Name        Variance Std.Dev.
##  Bottle   (Intercept) 0.002738 0.05233
##  Residual             0.006270 0.07918
## Number of obs: 6, groups:  Bottle, 3
##
## Fixed effects:
##              Estimate Std. Error t value
## (Intercept)   0.52000    0.05480    9.49
```

```
## Fraction0.8-2  4.70933    0.06465   72.84
##
## Correlation of Fixed Effects:
##             (Intr)
## Fractn0.8-2 -0.590
```

```
#drop function perform valid likelihood ratio tests
drop1(fit_Cpico, test = "Chisq")
```

```
## Single term deletions
##
## Model:
## C.uptake ~ Fraction + (1 | Bottle)
##          npar    AIC    LRT   Pr(Chi)
## <none>        -3.522
## Fraction    1 33.314 38.835 4.611e-10 ***
## ---
## Signif. codes:  0 '***' 0.001 '**' 0.01 '*' 0.05 '.' 0.1 ' ' 1
```

```
#it shows that 'fraction' significantly influences C uptake

#Report results
report(fit_Cpico)
```

```
## We fitted a linear mixed model (estimated using ML and nloptwrap optimizer) to
## predict C.uptake with Fraction (formula: C.uptake ~ Fraction). The model
## included Bottle as random effect (formula: ~1 | Bottle). The model's total
## explanatory power is substantial (conditional R2 = 1.00) and the part related
## to the fixed effects alone (marginal R2) is of 1.00. The model's intercept,
## corresponding to Fraction = 0.2-0.8, is at 0.52 (95% CI [0.28, 0.76], t(2) =
## 9.49, p = 0.011). Within this model:
##
##   - The effect of Fraction [0.8-2] is statistically significant and positive
## (beta = 4.71, 95% CI [4.43, 4.99], t(2) = 72.84, p < .001; Std. beta = 1.82,
## 95% CI [1.72, 1.93])
##
## Standardized parameters were obtained by fitting the model on a standardized
## version of the dataset. 95% Confidence Intervals (CIs) and p-values were
## computed using a Wald t-distribution approximation.
```

```
#Represent data predicted by the model vs real data
Fe_upt_pico$CPredicted <- exp(predict(fit_Cpico, re.form = NA))
```

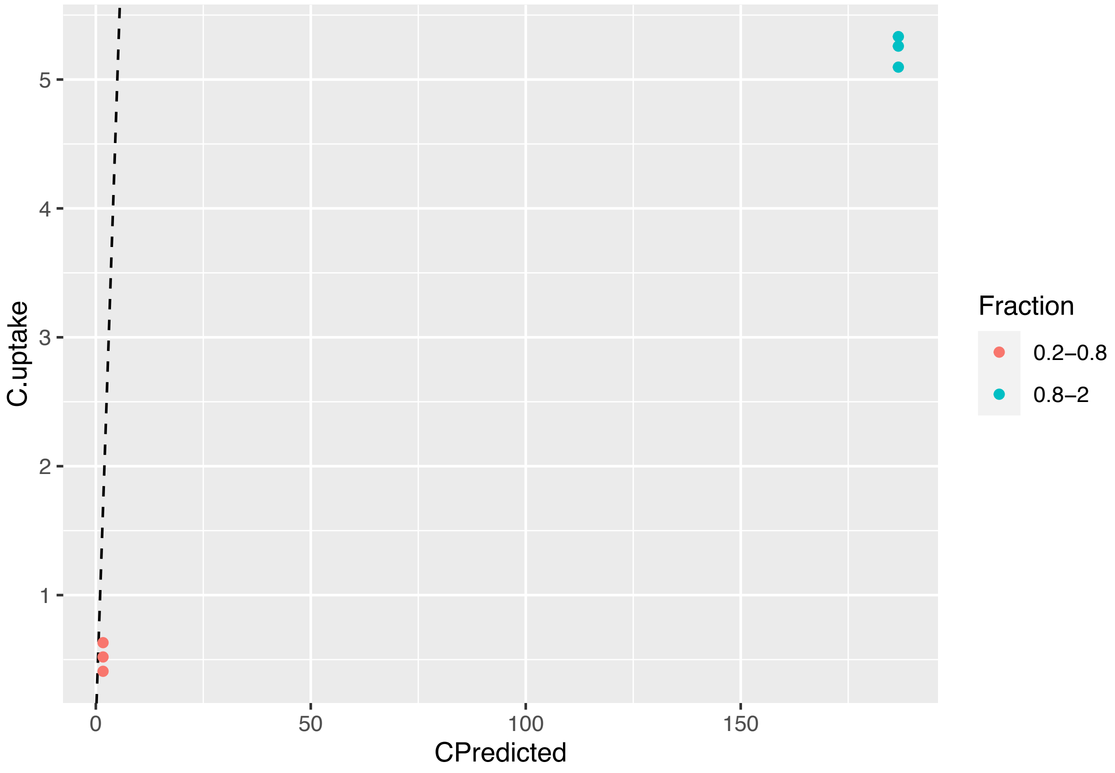

C.uptake
5
4
3
2
1
0
50
100
150
CPredicted
Fraction
0.2–0.8
0.8–2

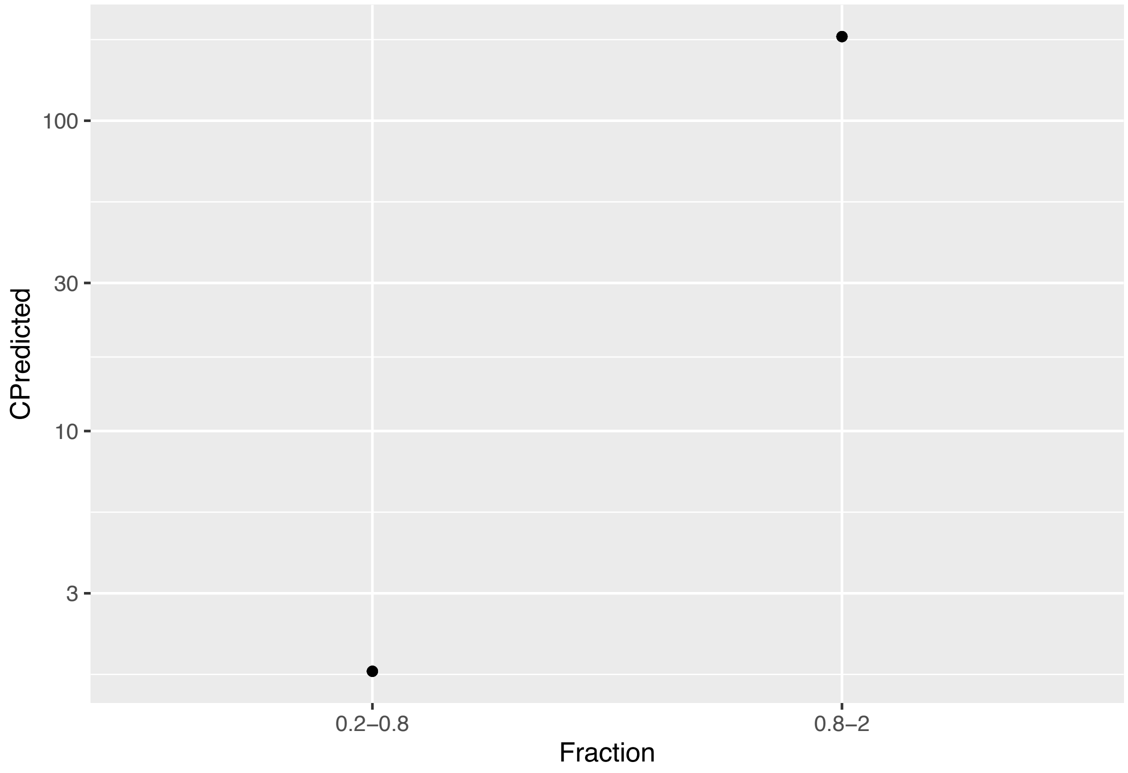


```
#Pairwise comparison
emmeans (fit_Cpico, pairwise ~ Fraction, adjust = "tukey")
```

```
## $emmeans
##  Fraction emmean     SE   df lower.CL upper.CL
##  0.2-0.8    0.52 0.0671 8.24    0.366    0.674
##  0.8-2      5.23 0.0671 8.24    5.075    5.383
##
## Degrees-of-freedom method: kenward-roger
## Confidence level used: 0.95
##
## $contrasts
##  contrast            estimate     SE  df t.ratio p.value
##  (0.2-0.8) - (0.8-2)    -4.71 0.0792 4.5 -59.475  <.0001
##
## Degrees-of-freedom method: kenward-roger
```

7 Fe uptake with pre-filtration and light

7.1 LMM test

```r
Fe_upt <- read.csv("Fe_uptake_filtrations_PL.csv")
```

```r
Fe_upt$ln_Fe <- log(Fe_upt$Fe.uptake.Cbm)   #data log-transformed
```

```r
#Plot using ggplot2
ggplot(Fe_upt) +
  geom_point(aes(x = Light, y = ln_Fe, col=Pre_filtration)) +
  facet_grid(Time ~ .) +
  scale_y_log10()
```

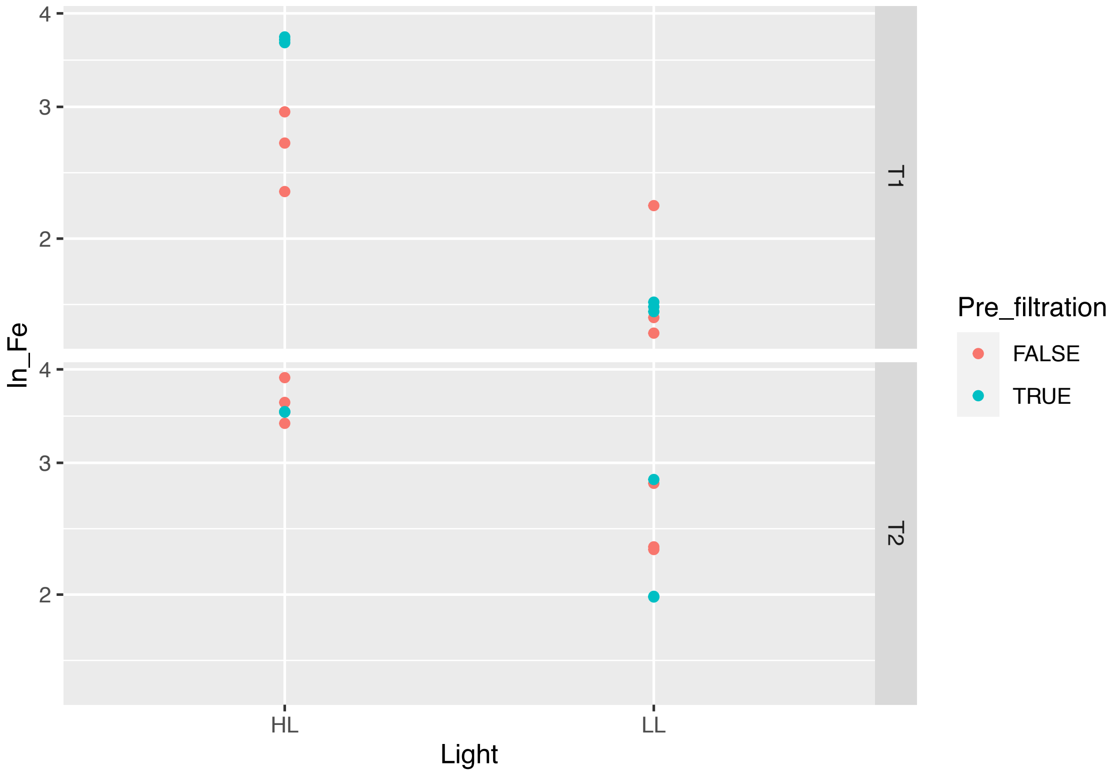


```r
#first model fit with all factors (Light, pre-filtration and time)
fit_Fe <- lmer(ln_Fe ~ Light + Pre_filtration + Time +
                 Light:Pre_filtration + Light:Time + Pre_filtration:Time + (1|Bottle),
               data = Fe_upt, na.action=na.fail, REML = FALSE)

summary(fit_Fe)
```

```
## Linear mixed model fit by maximum likelihood  ['lmerMod']
## Formula: ln_Fe ~ Light + Pre_filtration + Time + Light:Pre_filtration +
##     Light:Time + Pre_filtration:Time + (1 | Bottle)
##    Data: Fe_upt
##
##      AIC      BIC   logLik deviance df.resid
```

```
##     23.3     33.9     -2.7      5.3       15
##
## Scaled residuals:
##     Min      1Q  Median      3Q     Max
## -1.5786 -0.5444 -0.2866  0.4457  2.5509
##
## Random effects:
##  Groups   Name        Variance Std.Dev.
##  Bottle   (Intercept) 0.01233  0.1111
##  Residual             0.06174  0.2485
## Number of obs: 24, groups:  Bottle, 12
##
## Fixed effects:
##                            Estimate Std. Error t value
## (Intercept)                  2.7872     0.1487  18.740
## LightLL                     -1.1652     0.1977  -5.893
## Pre_filtrationTRUE           0.7638     0.1977   3.863
## TimeT2                       0.7091     0.1757   4.036
## LightLL:Pre_filtrationTRUE  -0.6273     0.2400  -2.613
## LightLL:TimeT2               0.2832     0.2029   1.396
## Pre_filtrationTRUE:TimeT2   -0.6131     0.2029  -3.022
##
## Correlation of Fixed Effects:
##             (Intr) LghtLL Pr_TRUE TimeT2 LLL:P_ LLL:TT
## LightLL     -0.665
## Pr_fltrTRUE -0.665  0.368
## TimeT2      -0.591  0.296  0.296
## LgLL:P_TRUE  0.403 -0.607 -0.607   0.000
## LghtLL:TmT2  0.341 -0.513  0.000  -0.577  0.000
## Pr_TRUE:TT2  0.341  0.000 -0.513  -0.577  0.000  0.000
```

```
#drop function perform valid likelihood ratio tests
drop1(fit_Fe, test = "Chisq")
```

```
## Single term deletions
##
## Model:
## ln_Fe ~ Light + Pre_filtration + Time + Light:Pre_filtration +
##     Light:Time + Pre_filtration:Time + (1 | Bottle)
##                     npar    AIC    LRT  Pr(Chi)
## <none>                   23.307
## Light:Pre_filtration    1 26.714 5.4067 0.020059 *
## Light:Time              1 23.112 1.8052 0.179084
## Pre_filtration:Time     1 28.256 6.9484 0.008389 **
## ---
## Signif. codes:  0 '***' 0.001 '**' 0.01 '*' 0.05 '.' 0.1 ' ' 1
```

```
#it shows that interactions between Light:Pre-filtrations and Pre-filtration:Time are significant
#non-significant interactions are removed from the model (here it's Light:Time)

#second model fit
fit_Fe2 <- lmer(ln_Fe ~ Light + Pre_filtration + Time + Light:Pre_filtration +
                  Pre_filtration:Time + (1|Bottle),
```

```r
                  data = Fe_upt, na.action=na.fail, REML = FALSE)
summary(fit_Fe2)
```

```
## Linear mixed model fit by maximum likelihood  ['lmerMod']
## Formula: ln_Fe ~ Light + Pre_filtration + Time + Light:Pre_filtration +
##     Pre_filtration:Time + (1 | Bottle)
##    Data: Fe_upt
##
##      AIC      BIC   logLik deviance df.resid
##     23.1     32.5     -3.6      7.1       16
##
## Scaled residuals:
##     Min      1Q  Median      3Q     Max
## -1.3264 -0.7265 -0.2797  0.7233  2.7497
##
## Random effects:
##  Groups   Name        Variance Std.Dev.
##  Bottle   (Intercept) 0.007321 0.08556
##  Residual             0.071765 0.26789
## Number of obs: 24, groups:  Bottle, 12
##
## Fixed effects:
##                             Estimate Std. Error t value
## (Intercept)                   2.7164     0.1428  19.027
## LightLL                      -1.0237     0.1697  -6.032
## Pre_filtrationTRUE            0.7638     0.2019   3.783
## TimeT2                        0.8506     0.1547   5.500
## LightLL:Pre_filtrationTRUE  -0.6273     0.2400  -2.613
## Pre_filtrationTRUE:TimeT2   -0.6131     0.2187  -2.803
##
## Correlation of Fixed Effects:
##             (Intr) LghtLL Pr_TRUE TimeT2 LLL:P_
## LightLL     -0.594
## Pr_fltrTRUE -0.707  0.420
## TimeT2      -0.542  0.000  0.383
## LgLL:P_TRUE  0.420 -0.707 -0.594   0.000
## Pr_TRUE:TT2  0.383  0.000 -0.542  -0.707  0.000
```

```r
#drop function perform valid likelihood ratio tests
drop1(fit_Fe2, test = "Chisq")
```

```
## Single term deletions
##
## Model:
## ln_Fe ~ Light + Pre_filtration + Time + Light:Pre_filtration +
##     Pre_filtration:Time + (1 | Bottle)
##                      npar    AIC    LRT Pr(Chi)
## <none>                    23.112
## Light:Pre_filtration    1 26.519 5.4067 0.02006 *
## Pre_filtration:Time     1 27.458 6.3454 0.01177 *
## ---
## Signif. codes:  0 '***' 0.001 '**' 0.01 '*' 0.05 '.' 0.1 ' ' 1
```

```r
#Report results
report(fit_Fe2)
```

```
## We fitted a linear mixed model (estimated using ML and nloptwrap optimizer) to
## predict ln_Fe with Light, Pre_filtration and Time (formula: ln_Fe ~ Light +
## Pre_filtration + Time + Light:Pre_filtration + Pre_filtration:Time). The model
## included Bottle as random effect (formula: ~1 | Bottle). The model's total
## explanatory power is substantial (conditional R2 = 0.89) and the part related
## to the fixed effects alone (marginal R2) is of 0.88. The model's intercept,
## corresponding to Light = HL, Pre_filtration = [?] and Time = T1, is at 2.72
## (95% CI [2.41, 3.02], t(16) = 19.03, p < .001). Within this model:
##
##   - The effect of Light [LL] is statistically significant and negative (beta =
## -1.02, 95% CI [-1.38, -0.66], t(16) = -6.03, p < .001; Std. beta = -1.24, 95%
## CI [-1.68, -0.80])
##   - The effect of Pre filtrationTRUE is statistically significant and positive
## (beta = 0.76, 95% CI [0.34, 1.19], t(16) = 3.78, p = 0.002; Std. beta = 0.92,
## 95% CI [0.41, 1.44])
##   - The effect of Time [T2] is statistically significant and positive (beta =
## 0.85, 95% CI [0.52, 1.18], t(16) = 5.50, p < .001; Std. beta = 1.03, 95% CI
## [0.63, 1.43])
##   - The effect of Light [LL] × Pre filtrationTRUE is statistically significant
## and negative (beta = -0.63, 95% CI [-1.14, -0.12], t(16) = -2.61, p = 0.019;
## Std. beta = -0.76, 95% CI [-1.38, -0.14])
##   - The effect of Pre filtrationTRUE × Time [T2] is statistically significant and
## negative (beta = -0.61, 95% CI [-1.08, -0.15], t(16) = -2.80, p = 0.013; Std.
## beta = -0.74, 95% CI [-1.30, -0.18])
##
## Standardized parameters were obtained by fitting the model on a standardized
## version of the dataset. 95% Confidence Intervals (CIs) and p-values were
## computed using a Wald t-distribution approximation.
```

```r
#Represent data predicted by the model vs real data
Fe_upt$Predicted <- exp(predict(fit_Fe2, re.form = NA))
```

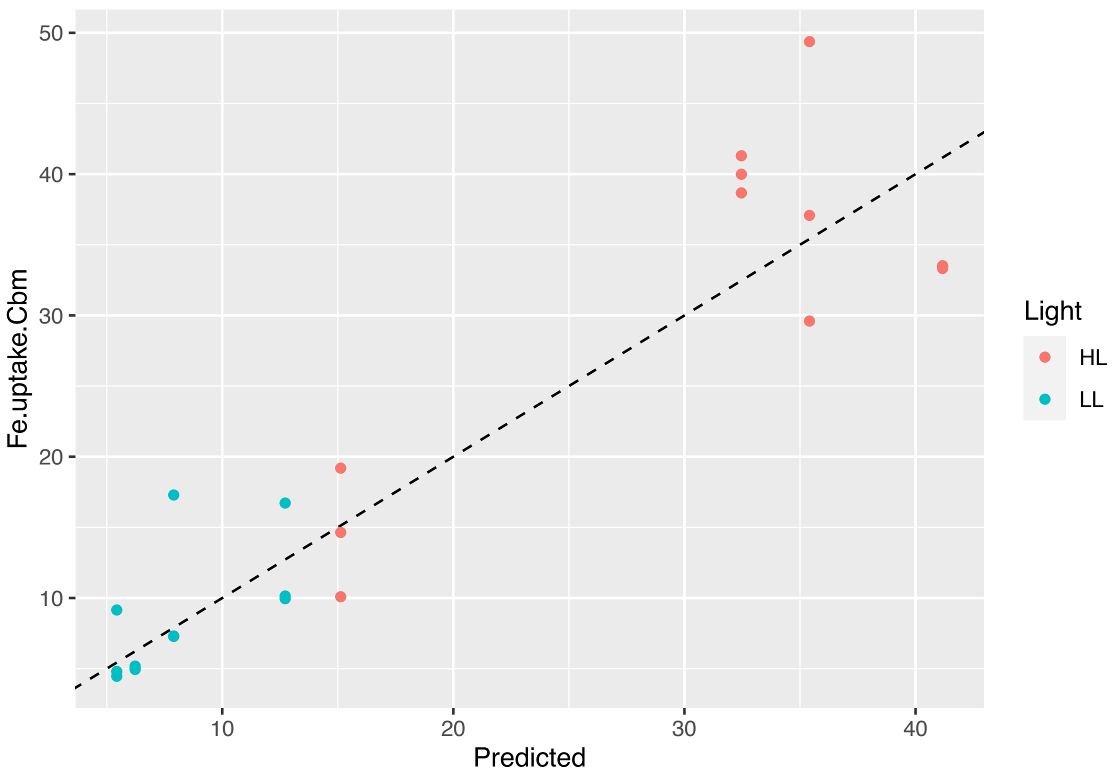

Fe.uptake.Cbm
50
40
30
20
10
10
20
30
40
Predicted
Light
HL
LL

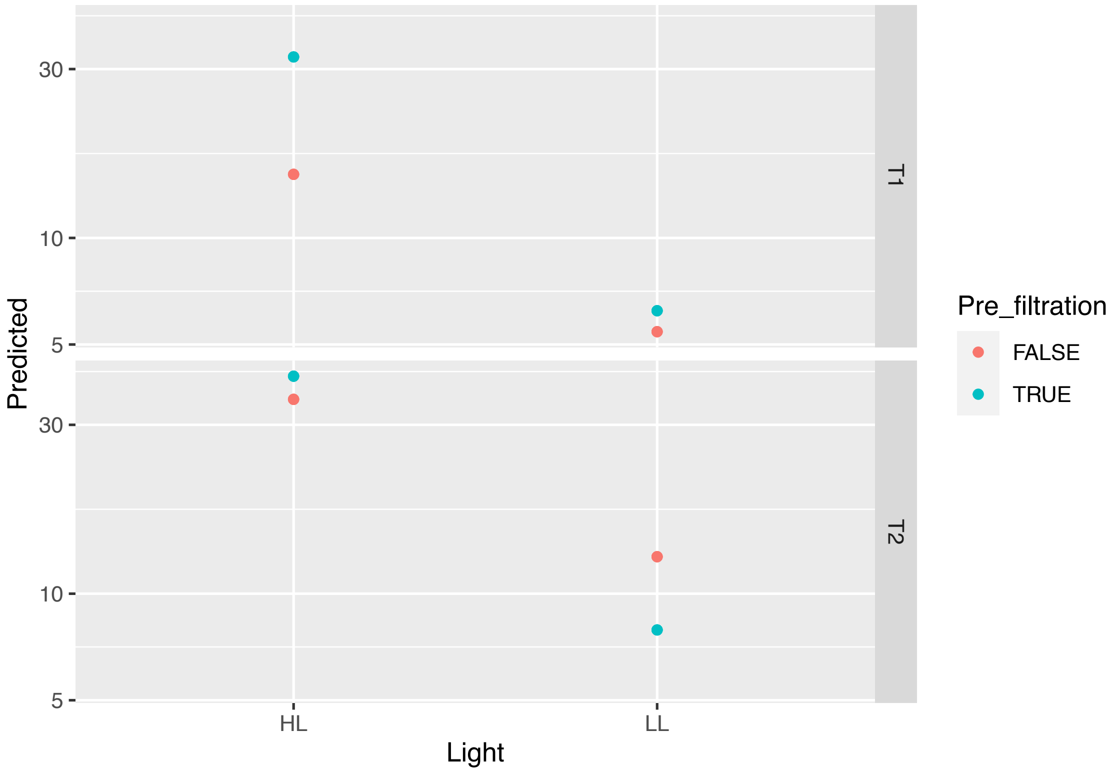


```r
#Pairwise comparison
emmeans (fit_Fe2, pairwise ~ Light + Pre_filtration + Time, adjust = "tukey")
```

```
## $emmeans
##  Light Pre_filtration Time emmean   SE   df lower.CL upper.CL
##  HL    FALSE          T1     2.72 0.17 29.7     2.37     3.06
##  LL    FALSE          T1     1.69 0.17 29.7     1.35     2.04
##  HL     TRUE          T1     3.48 0.17 29.7     3.13     3.83
##  LL     TRUE          T1     1.83 0.17 29.7     1.48     2.18
##  HL    FALSE          T2     3.57 0.17 29.7     3.22     3.91
##  LL    FALSE          T2     2.54 0.17 29.7     2.20     2.89
##  HL     TRUE          T2     3.72 0.17 29.7     3.37     4.06
##  LL     TRUE          T2     2.07 0.17 29.7     1.72     2.41
##
## Degrees-of-freedom method: kenward-roger
## Confidence level used: 0.95
##
## $contrasts
##  contrast                  estimate    SE   df t.ratio p.value
##  HL FALSE T1 - LL FALSE T1   1.0237 0.208 18.0   4.925  0.0022
##  HL FALSE T1 - HL TRUE T1   -0.7638 0.240 29.7  -3.184  0.0589
##  HL FALSE T1 - LL TRUE T1    0.8871 0.240 29.7   3.698  0.0174
##  HL FALSE T1 - HL FALSE T2  -0.8506 0.169 14.4  -5.021  0.0032
##  HL FALSE T1 - LL FALSE T2   0.1730 0.268 32.4   0.645  0.9978
##  HL FALSE T1 - HL TRUE T2   -1.0013 0.240 29.7  -4.174  0.0052
##  HL FALSE T1 - LL TRUE T2    0.6496 0.240 29.7   2.708  0.1598
```

```
##  LL FALSE T1 - HL TRUE T1   -1.7874 0.240 29.7  -7.450  <.0001
##  LL FALSE T1 - LL TRUE T1   -0.1365 0.240 29.7  -0.569  0.9990
##  LL FALSE T1 - HL FALSE T2  -1.8743 0.268 32.4  -6.989  <.0001
##  LL FALSE T1 - LL FALSE T2  -0.8506 0.169 14.4  -5.021  0.0032
##  LL FALSE T1 - HL TRUE T2   -2.0250 0.240 29.7  -8.441  <.0001
##  LL FALSE T1 - LL TRUE T2   -0.3741 0.240 29.7  -1.559  0.7695
##  HL TRUE T1 - LL TRUE T1     1.6509 0.208 18.0   7.943  <.0001
##  HL TRUE T1 - HL FALSE T2   -0.0869 0.240 29.7  -0.362  1.0000
##  HL TRUE T1 - LL FALSE T2    0.9368 0.240 29.7   3.905  0.0104
##  HL TRUE T1 - HL TRUE T2    -0.2376 0.169 14.4  -1.402  0.8426
##  HL TRUE T1 - LL TRUE T2     1.4134 0.268 32.4   5.271  0.0002
##  LL TRUE T1 - HL FALSE T2   -1.7378 0.240 29.7  -7.243  <.0001
##  LL TRUE T1 - LL FALSE T2   -0.7141 0.240 29.7  -2.977  0.0926
##  LL TRUE T1 - HL TRUE T2    -1.8885 0.268 32.4  -7.042  <.0001
##  LL TRUE T1 - LL TRUE T2    -0.2376 0.169 14.4  -1.402  0.8426
##  HL FALSE T2 - LL FALSE T2   1.0237 0.208 18.0   4.925  0.0022
##  HL FALSE T2 - HL TRUE T2   -0.1507 0.240 29.7  -0.628  0.9981
##  HL FALSE T2 - LL TRUE T2    1.5002 0.240 29.7   6.253  <.0001
##  LL FALSE T2 - HL TRUE T2   -1.1743 0.240 29.7  -4.895  0.0008
##  LL FALSE T2 - LL TRUE T2    0.4766 0.240 29.7   1.986  0.5070
##  HL TRUE T2 - LL TRUE T2     1.6509 0.208 18.0   7.943  <.0001
##
## Degrees-of-freedom method: kenward-roger
## P value adjustment: tukey method for comparing a family of 8 estimates
```
